# Is Guillain–Barré Syndrome Associated With COVID-19 Infection? A Systemic Review of the Evidence

**DOI:** 10.3389/fneur.2020.566308

**Published:** 2021-01-13

**Authors:** Auwal Abdullahi, Sevim Acaroz Candan, Melda Soysal Tomruk, Nuray Elibol, Olumide Dada, Steven Truijen, Wim Saeys

**Affiliations:** ^1^Department of Physiotherapy, Bayero University, Kano, Nigeria; ^2^Department of Physiotherapy and Rehabilitation Sciences, University of Antwerp, Antwerp, Belgium; ^3^Department of Physiotherapy and Rehabilitation, Faculty of Health Sciences, Ordu University, Ordu, Turkey; ^4^Department of Physiotherapy and Rehabilitation, Faculty of Health Sciences, Mehmet Akif University, Burdur, Turkey; ^5^Department of Physiotherapy and Rehabilitation, Faculty of Health Sciences, Ege University, Izmir, Turkey; ^6^Department of Physiotherapy, University of Ibadan, Ibadan, Nigeria

**Keywords:** COVID-19, Guillain Barre syndrome (GBS), electromyography, olfactory bulb, cytokines storms, reactive protein, physiotherapy, intravenous immunoglobulin

## Abstract

**Background:** There is emerging evidence that Guillain–Barré syndrome (GBS) may be associated with coronavirus disease 2019 (COVID-19) infection. The aim of this review was to investigate the strength of the evidence.

**Method:** The review was registered in PROSPERO (CDR42020184822). Three electronic databases, MEDLINE, PubMed, and Web of Science, and three preprint servers, MedRvix, ChemRvix, and BioRvix, were searched from December 2019 to 24th September 2020. Studies were included if they were on COVID-19 and of any design. Articles that are reviews or opinion were excluded. The selection process was carried out using EndNote and Rayyan software. The main outcomes in the study were study design, sample size, sex, age, overall GBS symptoms, other COVID-19 symptoms, comorbidity, timing between infection and the onset of neurological symptoms, CT, MRI, and EMG results. Methodological quality of the studies was assessed using the McMaster Critical Review Form. The collected data was analyzed using qualitative synthesis.

**Findings:** Fifty-one high-quality studies (mostly) consisting of 83 patients were included in the study. All of the patients (except in a very few) in the included studies had confirmed diagnosis of COVID-19. Similarly, the diagnosis of GBS was based on standard clinical, electrophysiological, and cerebrospinal fluid (CSF) criteria.

**Conclusion:** GBS may be associated with COVID-19, and therefore, testing for COVID-19 is recommended in patients presenting with GBS during this pandemic.

## Introduction

The novel coronavirus was first reported in Wuhan, China, in December 2019, and the world has since grappled under the effect of the coronavirus disease 2019 (COVID-19) pandemic with more than three million confirmed cases (1). The infection primarily affects the respiratory epithelium (2). However, evidence of its capability of affecting other cells, tissues, organs, and systems are currently emerging (3, 4). One of these systems is the nervous system (3). Although the mechanisms through which COVID-19 affects the nervous system are to date poorly understood, it is believed that direct infection injury, immune-mediated injury, systemic hypoxia as a result of severe pneumonia, and expression of angiotensin-converting enzyme 2 (ACE 2), the receptor for COVID-19 in the nervous system, may play a role (3, 5–9). This is because all the aforementioned mechanisms can cause damage to the nervous system and ultimately impair its functions (10). In addition, it is believed that intranasal inoculation of COVID-19 could cause damage to the olfactory epithelium and bulb (11). Furthermore, the virus can access the olfactory bulb *via* the peripheral neurons (12). This is because the olfactory neurons are directly exposed to the external environment at the sites of the dendritic nerve terminals (13). Thus, there could be spread of the virus through axonal transport as well as from the olfactory and trigeminal nerve endings in the nasal epithelium (14).

In addition, it was previously postulated that an outbreak of an infectious disease may trigger Guillain–Barré syndrome (GBS) (15). Consequently, one of the potential neurological complications during COVID-19 could be GBS. To buttress the above postulation, infections by viruses such as Zika, influenza, cytomegalo, and Epstein–Barr have been implicated in the pathogenesis of GBS (16, 17). Similarly, there is currently emerging evidence that COVID-19 is associated with GBS (18, 19). In general, GBS is clinically characterized by the absence of reflexes and increased concentration of cerebrospinal fluid protein that progress very rapidly (20). Neurophysiologically, what are seen are small action potentials, prolonged distal motor latency, delayed F waves, and conduction block (21). The aim of this systematic review is to summarize the evidence on the association between GBS and COVID-19 infection.

## Methods

### Strategy and Selection Criteria

The study design was a systematic review whose protocol was registered in PROSPERO, a registry for systematic reviews owned and managed by the University of York in the United Kingdom. The registration number is CDR42020184822. In addition, the study was carried out accordance with the criteria set out in the Preferred Reporting Items for Systematic Reviews and Meta-Analyses (PRISMA) guideline (22).

Studies of any design were included if they were on COVID-19 and had reported GBS as a neurological complication of the disease. In addition, the included studies were only those published in English from December 2019 to 24th September 2020. Reviews and opinion articles were excluded from the study.

Three electronic databases, MEDLINE, PubMed, and Web of Science, and three electronic preprint servers, MedRvix, ChemRvix, and BioRvix, were searched from December 2019 to 24th September 2020. Similarly, the lists of references of the included studies were screened and Google search was also carried out for any relevant papers. Coronavirus, signs and symptoms, and Guillain–Barré syndrome were used as some of the key search terms. However, during the search, these key search terms were modified according to the requirements of each database and appropriate Boolean operators were used to combine the terms. The strategy was adopted from our previous systematic review on neurological and musculoskeletal features of COVID-19 (23). The search was carried out by one of the reviewers (MST). See Appendix 1 for the details of the search strategy used in MEDLINE.

Duplicates were removed using EndNote and Rayyan software. Similarly, the Rayyan software was used for the selection of the eligible studies (24). Two reviewers (SAC and NE), researchers who have experience in doing systematic review, performed the study selection. In case of any disagreement in the selection process, consensus discussion and/or a third reviewer (MST) was used to resolve it. The selection was done according to the study inclusion and exclusion criteria.

### Data Analysis

A data extraction form was used to extract the data from the included studies. The data extracted include the study title; study design; authors; year of publication; country; sample size; sex; age; overall GBS symptoms the patients present with; other COVID-19 symptoms; comorbidity; timing between infection and the onset of neurological symptoms; treatment received; diagnostic criteria for GBS and COVID-19; other examinations such as CT, MRI, and EMG carried out to confirm the presence of GBS; and mortality status. Data on electrophysiological subtypes were also extracted. The data extraction was carried out by SAC, NE, and AA independently and consensus was achieved through discussion.

The methodological quality of the included studies was appraised independently by AA and OD and confirmed by WS and ST using the Modified McMaster Critical Review Form. The form consists of 17 items that assesses study purpose, literature review, study design, study sample, reliability, and validity of the study outcomes, interventions given, results, and conclusions (25). Each of the items is scored on a four-point scale, represented by yes, no, not addressed, and not applicable. When the answer to a particular item is no or not addressed, a score of zero is allocated, and when it is yes, a score of one is allocated. However, no score is allocated when the item is not applicable to a particular study design such as case reports or observational studies. The total scores from the appraisal can then be classified as poor, fair, good, or excellent quality when they are 1/4 or less, ≤ 2/4, ≥2/4 but ≤ 3/4 and >3/4–4/4 of the total score, respectively. When there were any disputes between the assessors, it was resolved through consensus discussion and/or through contacting a third reviewer. Similarly, the National Health and Medical Research Council's (NHMRC) evidence hierarchy was used to determine the level of evidence (26).

Qualitative synthesis which involves narrative synthesis of the characteristics and findings of the included studies was used for the data analysis. Some of the results of the synthesis were reported in the form of sum, frequency and percentage, mean and standard deviation, study flow chart, and summary tables.

### Role of the Funding Source

There was no funding source for this study. However, any information about access to data and responsibility for submission can be directed to the corresponding author (AA).

## Result

**Figure 1:**
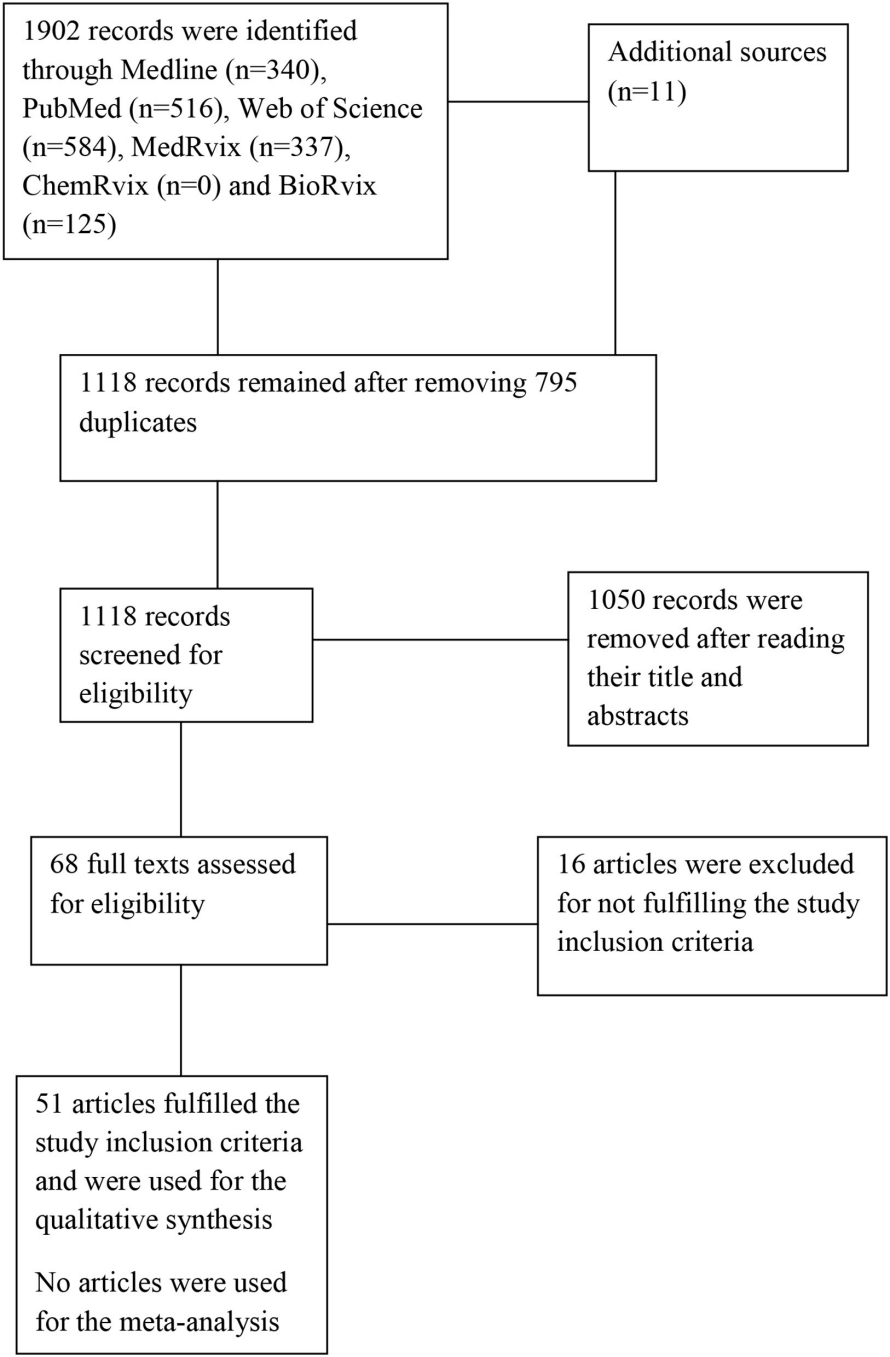
The study flowchart.

The search yielded a total of 1,913 hits in which only 51 articles were eligible for inclusion in the review (18, 19, 27–75). See Figure 1 for the PRISMA flowchart.

The total number of participants in the included studies was 83 in which 60 were male and 23 were female. However, five out of the total number of participants were children (56–58, 62, 68). Consequently, the age range of the patients was between 11 and 94 years.

The countries where the studies were carried out were Italy (18, 27, 30–32, 39, 40, 60, 65, 71, 75), China (21), Iran (22, 62, 64, 68, 69), Morocco (38), Netherlands (41), Turkey (42), Canada (43), Austria (47), Switzerland (48, 54), Spain (34, 35, 37, 45, 46, 52, 73), United States (19, 33, 49, 59, 66), France (36, 44, 50), UK (53, 74), Germany (55, 61, 70), Tanzania (56), Brazil (57), Saudi Arabia (58), and Sudan (72). However, in a few of the studies and the patients, GBS symptoms preceded the symptoms of COVID-19 infection with a range of 7–10 days (27, 28, 44). In addition, in some of the studies and the patients, it could not be determined whether COVID-19 symptoms preceded GBS or vice versa (42, 43, 47, 57, 60, 62, 71).

All the studies used reverse transcription polymerase chain reaction (RT-PCR) for the diagnosis of COVID-19. In some of the studies, nasal swab RT-PCR tested negative (31, 39, 45, 47, 48). However, in some of the studies, IgG was positive using the ELISA technique (48, 57, 67, 75).

Some of the studies tested for antiganglioside antibodies (18, 35–37, 39–41, 44, 45, 48–50, 61, 65–67, 71, 73, 75). The test was positive for the antibodies GD1b-IgG, IgM for GM2 and GD3, and a weak IgG band for GT1b and GD1a in some patients in only five studies (35, 37, 66, 67, 71). However, the GM1 antibody was in the equivocal range in one study (51). In addition, in all the studies in which real-time polymerase chain reaction assay of the cerebrospinal fluid (CSF) was performed, COVID-19 was only detected in the CSF in one study (47).

COVID-19 symptoms the patients presented with were dyspnea (19, 27, 29, 46, 50, 54, 63, 64, 68, 69, 75), pharygitis (18, 69, 72), fever (18, 19, 27, 28, 30–37, 39, 40, 42–44, 46, 47, 50, 51, 56, 58, 59, 61, 64–67, 69–72, 75), anosmia or hyposmia (18, 31, 40, 44, 47, 65, 68, 75), cough (18, 19, 25, 28–30, 32, 34, 35, 37–41, 44–48, 50, 53, 55, 56, 58, 59, 61, 63–65, 68–71, 75), ageusia or dysgeusia (18, 31, 39, 40, 44, 47, 55, 59, 65, 67, 75), chest pain (28, 54), diarrhea (19, 28, 35, 44, 45, 49, 50, 52, 54, 59, 71), headache (31, 35, 47, 53, 72), myalgia (31, 37, 44, 47, 48, 53, 54, 58, 62, 63, 67, 68), fatigue (32, 47, 48, 59, 72), rhinorrhea (33), odynophagia (33, 45, 54), chills (33, 49, 51, 54), night sweats (33), malaise (35, 53), anorexia (49), asthenia (44, 67), unspecified upper respiratory symptoms (52, 58, 66), low back pain (35), nausea (54, 59), vomiting (54), vasovagal syncope (54), arthralgia (54), sinonasal congestion (59), dizziness (62), and gastrointestinal symptoms (75).

Magnetic resonance imaging (MRI) of the brain showed normal findings in some of the studies (28, 30, 34, 40, 41, 57, 65). However, in some of the studies, it revealed enhancement of some cranial nerves such as the facial nerve (CN VII) bilaterally, CN III, CN V, and CN VI (18, 42, 43, 51, 59). For the spine, MRI showed normal findings in some of the studies (18, 19, 31, 33, 51, 57), mild herniation of two intervertebral discs, and enhancement or thickening and hyperintensity of brachial, caudal, and lumbosacral nerves and roots in some of the patients (18, 29, 44, 54, 58, 70). However, brain CT showed normal findings in some of the patients (27, 34, 43, 49, 50).

Lung computed tomography (CT) revealed ground-glass opacities in one or both lungs (27, 29–33, 36, 38, 42, 43, 47, 50, 52, 68, 69, 71, 72), bilateral pleural effusion and bilateral basilar opacities (29), diffused consolidation (27, 29, 42, 50, 72), patchy subsegmental faint opacifications with an atelectasis in the lingula (58), peri-bronchovascular thickening (71), bilateral interstitial infiltrates (70, 71), mild lung involvement (46), and normal findings (60).

Chest X-ray revealed diffuse heterogeneous infiltration in both lungs (43), mild bibasilar atelectasis and patchy consolidations (49), bilateral basilar opacifications (56), bilateral paracardiac and basal veiling opacities (58), patchy bilateral air space opacities without lobar consolidation (59), and no conspicuous findings (61).

Chest auscultation revealed bilateral diffuse crackles consistent with pneumonia (43, 50, 69) and bilateral crepitations to the mid-zones on lung auscultation (53).

The laboratory investigations showed elevated level of interleukin-6 and interleukin-8 (31, 60, 75), raised creatinine level (29), elevated level of creatine kinase (64), decreased creatinine level (49), raised creatinine phosphokinaselevel (60, 61), increased level of D-dimer (43, 46, 58), thrombocytopenia (46, 54), high level of fibrinogen (46), elevated brinogen level (47), elevated lactate level (63), increased lactate dehydrogenase level (47, 60), lymphocytosis (43, 68), raised C-reactive protein level (39, 42, 44, 47, 53, 60, 63, 64, 68), eosinopenia (38), leucopenia (54, 56, 61), lymphopenia (42, 53, 59, 64, 68), lymphocytopenia (38, 40, 54, 60), thrombocythemia (53), increased erythrocyte sedimentation rate (47, 64, 68), elevated glucose level (64), leukocytosis (40, 49), deceased potassium level (49), raised alanine transaminase level (49), and raised glutamic oxaloacetic transaminase and glutamic pyruvic transaminase (60).

Similarly, neurophysiological tests indicated abnormality in some of the studies.

Comorbidities reported in the studies were diabetes mellitus (22, 37, 50, 54, 64), *Clostridium difficile* colitis (23), hypertension (27, 31, 32, 37, 49, 53, 59, 62, 73), abdominal aortic aneurysm (27), lung cancer (27), hyperlipidemia (32), restless leg syndrome (32), chronic back pain (32), coronary artery disease (49), alcohol abuse (49), rheumatoid arthritis (38), left eye strabismus (51), prediabetes (59), class 1 obesity (59), and follicular lymphoma (73). See Supplementary Table 1 for the characteristic of the included studies.

Most of the studies are level VI evidence studies; only two studies are level IV evidence studies (71, 74). However, most of the studies had excellent methodological qualities. See Table 1 for the level of evidence and the methodological quality of the included studies.

**Table 1:** Levels of evidence and methodological quality of the included studies.

**References**	**Design**	**Level of evidence**	**1**	**2**	**3**	**4**	**5**	**6**	**7**	**8**	**9**	**10**	**11**	**12**	**13**	**14**	**15**	**16**	**17**	**Total score**
Alberti et al. (27)	Case report	IV	Yes	Yes	Yes	NA	NA	NA	Yes	Yes	Yes	Yes	NA	NA	NA	NA	NA	NA	Yes	8/8
Zhao et al. (28)	Case report	IV	Yes	No	Yes	NA	NA	NA	Yes	Yes	Yes	No	NA	NA	NA	NA	NA	NA	Yes	6/8
Sedaghat and Karimi (29)^2^	Case report	IV	Yes	Yes	Yes	NA	NA	NA	Yes	Yes	Yes	No	NA	NA	NA	NA	NA	NA	Yes	7/8
Virani et al. (19)	Case report	IV	Yes	Yes	Yes	NA	NA	NA	Yes	Yes	Yes	Yes	NA	NA	NA	NA	NA	NA	Yes	8/8
Toscano et al. (18)	Case series	IV	Yes	No	Yes	NA	NA	NA	Yes	Yes	Yes	Yes	NA	NA	NA	NA	NA	NA	Yes	7/8
Padroni et al. (30)	Case report	IV	Yes	Yes	Yes	NA	NA	NA	Yes	Yes	Yes	Yes	NA	NA	NA	NA	NA	NA	Yes	8/8
Riva et al. (31)	Case report	IV	Yes	Yes	Yes	NA	NA	NA	Yes	Yes	Yes	Yes	NA	NA	NA	NA	NA	NA	Yes	8/8
Ottaviani et al. (32)	Case report	IV	Yes	Yes	Yes	NA	NA	NA	Yes	Yes	Yes	Yes	NA	NA	NA	NA	NA	NA	Yes	8/8
Rana et al. (33)	Case report	IV	Yes	Yes	Yes	NA	NA	NA	Yes	Yes	Yes	Yes	NA	NA	NA	NA	NA	NA	Yes	8/8
Caamaño and Beato (34)	Case report	IV	Yes	Yes	Yes	NA	NA	NA	Yes	Yes	Yes	Yes	NA	NA	NA	NA	NA	NA	Yes	8/8
Gutiérrez-Ortiz et al. (35)	Case series	IV	Yes	Yes	Yes	NA	NA	NA	Yes	Yes	Yes	Yes	NA	NA	NA	NA	NA	NA	Yes	8/8
Camdessanche et al. (36)	Case report	IV	No	No	Yes	NA	NA	NA	Yes	Yes	Yes	Yes	NA	NA	NA	NA	No	NA	Yes	5/8
Diez-Porras et al. (37)	Case report	IV	Yes	Yes	Yes	NA	NA	NA	Yes	Yes	Yes	Yes	NA	NA	NA	NA	No	NA	Yes	6/9
El Otmani et al. (38)	Case report	IV	Yes	Yes	Yes	NA	NA	NA	Yes	Yes	Yes	Yes	NA	NA	NA	NA	No	NA	Yes	8/9
Zito et al. (39)	Case report	IV	Yes	Yes	Yes	NA	NA	NA	Yes	Yes	Yes	Yes	NA	NA	NA	NA	NA	NA	Yes	8/8
Assini et al. (40)	Case series	IV	Yes	No	Yes	NA	NA	NA	Yes	Yes	Yes	Yes	NA	NA	NA	NA	NA	NA	Yes	7/8
Kilinc et al. (41)	Case report	IV	Yes	Yes	Yes	NA	NA	NA	Yes	Yes	Yes	Yes	NA	NA	NA	NA	NA	NA	Yes	8/8
Chan et al. (43)	Case report	IV	Yes	Yes	Yes	NA	NA	NA	Yes	Yes	Yes	Yes	NA	NA	NA	NA	NA	NA	Yes	8/8
Bigaut et al. (44)	Case series	IV	Yes	Yes	Yes	NA	NA	NA	Yes	Yes	Yes	No	NA	NA	NA	NA	Yes	NA	Yes	8/9
Reyes-Bueno et al. (45)	Case report	IV	Yes	Yes	Yes	NA	NA	NA	Yes	Yes	Yes	No	NA	NA	NA	NA	NA	NA	Yes	7/8
Marta-Enguita et al. (46)	Case report	IV	Yes	Yes	Yes	NA	NA	NA	No	Yes	Yes	Yes	NA	NA	NA	NA	NA	NA	Yes	7/8
Helbok et al. (47)	Case series	IV	Yes	Yes	Yes	NA	NA	NA	Yes	Yes	Yes	Yes	NA	NA	NA	NA	NA	NA	Yes	8/8
Coen et al. (48)	Case report	IV	No	No	Yes	NA	NA	NA	Yes	Yes	Yes	Yes	NA	NA	NA	NA	No	NA	Yes	6/8
Su et al. (49)	Case report	IV	Yes	Yes	Yes	NA	NA	NA	Yes	Yes	Yes	Yes	NA	NA	NA	NA	Yes	NA	Yes	9/9
Arnaud et al. (50)	Case report	IV	Yes	Yes	Yes	NA	NA	NA	Yes	Yes	Yes	Yes	NA	NA	NA	NA	Yes	NA	Yes	8/9
Lantos et al. (51)	Case report	IV	Yes	Yes	Yes	NA	NA	NA	No	No	No	No	NA	NA	NA	NA	No	NA	Yes	4/9
Velayos Galán et al. (52)	Case report	IV	Yes	Yes	Yes	NA	NA	NA	Yes	Yes	Yes	Yes	NA	NA	NA	NA	Yes	NA	Yes	9/9
Webb et al. (53)	Case report	IV	Yes	Yes	Yes	NA	NA	NA	Yes	Yes	Yes	Yes	NA	NA	NA	NA	Yes	NA	Yes	9/9
Lascano et al. (54)	Case series	IV	Yes	Yes	Yes	NA	NA	NA	Yes	Yes	Yes	Yes	Yes	NA	NA	NA	Yes	NA	Yes	9/9
Scheidl et al. (55)	Case report	IV	Yes	Yes	Yes	NA	NA	NA	Yes	Yes	Yes	Yes	NA	NA	NA	NA	Yes	NA	Yes	9/9
Manji et al. (56)	Case report	IV	Yes	Yes	Yes	NA	NA	NA	No	No	No	No	Yes	NA	NA	NA	No	NA	Yes	5/9
Frank et al. (57)	Case report	IV	Yes	Yes	Yes	NA	NA	NA	Yes	Yes	Yes	Yes	NA	NA	NA	NA	NA	NA	Yes	8/8
Khalifa et al. (58)	Case report	IV	Yes	Yes	Yes	NA	NA	NA	Yes	Yes	Yes	Yes	NA	NA	NA	NA	Yes	NA	Yes	9/9
Bracaglia et al. (60)	Case report	IV	Yes	Yes	Yes	NA	NA	NA	Yes	Yes	Yes	Yes	NA	NA	NA	NA	Yes	NA	Yes	8/8
Lampe et al. (61)	Case report	IV	Yes	Yes	Yes	NA	NA	NA	Yes	Yes	Yes	Yes	NA	NA	NA	NA	Yes	NA	Yes	9/9
Paybast et al. (62)	Case series	IV	Yes	Yes	Yes	NA	NA	NA	Yes	Yes	Yes	Yes	NA	NA	NA	NA	Yes	NA	Yes	9/9
Tiet and AlShaik (63)	Case report	IV	Yes	Yes	Yes	NA	NA	NA	Yes	Yes	Yes	Yes	NA	NA	NA	NA	No	NA	Yes	8/9
Farzi et al. (64)	Case report	IV	Yes	Yes	Yes	NA	NA	NA	Yes	Yes	Yes	Yes	NA	NA	NA	NA	No	NA	Yes	8/9
Manganotti et al. (75)^1^	Case report	IV	Yes	Yes	Yes	NA	NA	NA	No	No	No	No	NA	NA	NA	NA	Yes	NA	Yes	5/9
Chan et al. (43)^2^	Case series	IV	Yes	Yes	Yes	NA	NA	NA	Yes	Yes	Yes	No	NA	NA	NA	NA	No	NA	No	6/9
Naddaf et al. (67)	Case report	IV	Yes	Yes	Yes	NA	NA	NA	Yes	Yes	Yes	No	NA	NA	NA	NA	Yes	NA	Yes	8/9
Mozhdehipanah et al. (68)	Case series	IV	Yes	Yes	Yes	NA	NA	NA	Yes	Yes	Yes	No	NA	NA	NA	NA	No	NA	Yes	7/9
Ebrahimzadeh et al. (69)	Case series	IV	Yes	Yes	Yes	NA	NA	NA	Yes	Yes	Yes	No	NA	NA	NA	NA	No	NA	Yes	7/9
Pfefferkorn et al. (70)	Case report	IV	Yes	Yes	Yes	NA	NA	NA	Yes	Yes	Yes	No	NA	NA	NA	NA	No	NA	Yes	7/9
Gigli et al. (71)	Case control	III-3	Yes	Yes	Yes	NA	NA	NA	NA	Yes	Yes	No	NA	NA	NA	NA	Yes	NA	Yes	8/9
Sidig et al. (72)	Case report	IV	Yes	Yes	Yes	NA	NA	NA	Yes	Yes	Yes	Yes	NA	NA	NA	NA	Yes	NA	Yes	9/9
Fernández-Domínguez et al. (73)	Case report	IV	Yes	Yes	Yes	NA	NA	Yes	Yes	Yes	Yes	No	NA	NA	NA	NA	No	NA	Yes	7/9
Paterson et al. (74)	Cohort study	III-2	Yes	Yes	No	NA	NA	NA	No	No	No	No	NA	NA	NA	NA	No	No	No	2/10
Manganotti (75)	Case series	IV	Yes	Yes	Yes	NA	NA	NA	Yes	Yes	Yes	Yes	NA	NA	NA	NA	Yes	NA	Yes	9/9

## Discussion

Eleven articles with good and excellent methodological quality, consisting of a total number of 16 patients, were included in the review. In the reviewed studies, COVID-19 infection was confirmed using RT-PCR. GBS was confirmed using clinical examinations including muscle strength testing, neurophysiological and laboratory examinations, and CSF analysis, all of which showed abnormality consistent with the diagnostic criteria for GBS (76). The coexistence of GBS in these patients is not surprising since infections by Zika, influenza, cytomegalo, and Epstein–Barr viruses have been implicated in the etiology of GBS (16, 17). Consequently, it was hypothesized that GBS may be triggered especially during an outbreak of an infectious illness (15).

Although the mechanisms through which COVID-19 infection affects the nervous system are still poorly understood, there are some theoretical mechanisms through which infection is believed to injure the nervous system. One of the theories is that infectious agents such as viruses can get into the nervous system through blood circulation and/or retrograde neuronal pathways and infect the peripheral nerves (6). These have, however, not yet been reported in patients with COVID-19 as scientists are still working to understand its pathogenesis (77). Secondly, severe pneumonia, one of the classic symptoms of COVID-19, can result in systemic hypoxia that can deprive the nerves of oxygen and vital nutrients which may eventually result in the accumulation of toxic substances that are capable of damaging neurons (7, 10). Thirdly, the special affinity COVID-19 has for ACE 2 which is also noted to be present in the nervous system, the skeletal muscles, other tissues, and organs may as well serve as the reason for the neuronal pathology such as GBS seen in people with the disease (9, 78).

In addition, the neuronal damage may be caused by immune response-related injury. This is because, in response to an infection, levels of inflammatory cytokines such as the interleukin- 6 are raised. Similarly, activities of T lymphocytes, macrophages, and endothelial cells also increase. These can result in vascular leakage, activation of complement and coagulation cascade, and eventually end organ damage (5).

Similarly, in the reviewed studies, there were reports of impaired arterial blood gases indicating severe hypoxia, raised white cell counts, impaired erythrocyte sedimentation rate, and increased CSF protein levels, which are markers of pathology that can result in neuronal injury. Therefore, it is possible that GBS in patients with COVID-19 is caused by a number of different mechanisms including immune-mediated injury. However, in two of the studies, GBS symptoms preceded the COVID-19 symptoms (27, 28). This should be noted for the early diagnosis of COVID-19, and any patients presenting with GBS should be evaluated for the disease especially during the pandemic. This is consistent with the reports of many studies as patients with COVID-19 may not present with the classical symptoms of the disease such as fever and cough at the early stage, but may present with other symptoms such as anosmia and impaired taste sensation (3, 79).

Although in one of the reviewed studies, the participant had ataxia, it is difficult to say this was caused by the direct affectation of the cerebellum. This is because the cerebellum has not been reported to have ACE 2 receptors (80). This review has some strengths. Firstly, the comorbidities reported in the studies are not known to cause GBS. Secondly, the reviewed studies had confirmatory diagnosis of COVID-19 in the patients. Thirdly, outcomes such as from neurophysiological and clinical examinations and laboratory investigations were used in the diagnosis of GBS. These strengthened our claims for the association or relationship between COVID-19 infection and GBS. However, one of the limitations of the review is that, in most of the studies, the recovery outcomes of the patients were not clearly reported. This can undermine the quality of the reports. In addition, the reviewed studies were only those published in English. Therefore, it is possible that we missed very relevant information from studies published in other languages.

## Conclusion

COVID-19 infection affects the nervous system and can trigger GBS since there is consistency in the findings of the reviewed studies. Thus, patients presenting with GBS should be evaluated for COVID-19 as soon as possible especially during this pandemic.

## Panel: Research in Context

### Evidence Before This Study

There has been speculation on COVID-19 potential to affect the nervous system. In particular, there were few case reports on patients with COVID-19 presenting with Guillain–Barré syndrome. Three electronic databases, MEDLINE, PubMed, and Web of Science, and three preprint servers, MedRvix, ChemRvix, and BioRvix, were searched from December 2019 to 24th September 2020. Studies were included if they were on COVID-19 and of any design. Reviews and opinion articles were excluded. The key search terms used were coronavirus, signs and symptoms, and Guillain–Barré syndrome. Methodological quality of the studies was assessed using the McMaster Critical Review Form.

### Added Value of This Study

The study found converging evidence from different studies reporting on Guillain–Barré syndrome in patients with COVID-19.

### Implications of All the Available Evidence

Patients with COVID-19 may present with Guillain–Barré syndrome. Therefore, any patient presenting with this syndrome should be evaluated for COVID-19. This will help with the early diagnosis, treatment plan, and rehabilitation to help curb the spread of the disease.

## Data Availability Statement

The original contributions presented in the study are included in the article/supplementary materials, further inquiries can be directed to the corresponding author.

## Author Contributions

MS searched the literature. SC and NE selected the studies with input from MS and AA extracted the study data with input from SC and NE. AA and OD assessed the methodological quality of the studies with input from SC, WS, and ST. AA did the data analysis and interpretation and drafted the manuscript with input from SC, WS, and ST. SC, MS, NE, OD, WS, and ST critically reviewed the drafted manuscript. All the authors approved the manuscript for submission and contributed in designing the study.

## Conflict of Interest

The authors declare that the research was conducted in the absence of any commercial or financial relationships that could be construed as a potential conflict of interest.

## Appendix 1

**Table:**

1.	exp Coronavirus/
2.	Coronavirus Infections/
3.	covid 2019.mp.
4.	SARS2.mp.
5.	SARS-CoV-2.mp.
6.	severe acute respiratory syndrome coronavirus 2.mp.
7.	coronavirus infection.mp.
8.	severe acute respiratory pneumonia outbreak.mp.
9.	novel cov.mp.
10.	2019ncov.mp.
11.	sars cov2.mp.
12.	cov2.mp.
13.v	ncov.mp.
14.	covid-19.mp.
15.	covid19.mp.
16.	Coronaviridae/
17.	corona virus.mp.
18.	1 or 2 or 3 or 4 or 5 or 6 or 7 or 8 or 9 or 10 or 11 or 12 or 13 or 14 or 15 or 16 or 17
19.	“Signs and Symptoms”/
20.	(sign? adj2 symptom*).tw.
21.	(sign? or symptom* or complain*).tw.
22.	(clinical adj3 (manifestation? or feature? or finding? or aspect? or marker?)).tw.
23.	(presenting adj3 (feature? or finding? or factor?)).tw.
24.	presentation?.tw.
25.	(physical adj3 (manifestation? or characteristic? or feature? or finding?)).tw.
26.	GuillainBarre syndrome
27.	19 or 20 or 21 or 22 or 23 or 24 or 25 or 26
