# Supplementary material for: Is Guillain–Barré Syndrome Associated With COVID-19 Infection? A Systemic Review of the Evidence
Source: Front Neurol. 2021 Jan 13;11:566308. doi: 10.3389/fneur.2020.566308 (PMC7838680; doi:10.3389/fneur.2020.566308)
Supplement: Supplementary file 1 [file Table_1.docx]

**Supplementary Table 1: Characteristics of the Included Studies**

| **Study** | **Sample size (number of male patients)** | **Age**  **(years)** | **GBS symptoms** | **GBS sub-type** | **Timing between Covid-19 symptoms onset and GBS onset (days)** | **Treatments received** | **CSF Examination** | **Electrophysiology** | **Outcome** |
| --- | --- | --- | --- | --- | --- | --- | --- | --- | --- |
| Sedaghat & Karim (2020) | 1 (1) | 65 | Acute progressive symmetric ascending quadriparesis (Medical Research Council (MRC) scale of 2/5 in proximal, 3/5 in distal of the upper extremities and 1/5 in proximal, 2/5 in distal of the lower extremities), Bilateral facial paresis (House–Brackmann grade 3), absent DTR, reduction in the vibration and fine touch sensation distal to the ankle joints | AMAN | Covid-19 preceded GBS by 11 days | Hydroxychloroquine, Lopinavir/Ritonavir (LPV/RTV) and Azithromycin.  0.40g /kg/ day intravenous  Immunoglobulin for five days for GBS. | Nil | Delayed or not evocable distal latency, decreased amplitude, decreased or not evocable conduction velocity and absent F waves in distal extremities nerves. | No information the outcome of GBS. |

**Key**: DTR= deep tendon reflex, CSF=cerebrospinal fluid, AMAN=Acute motor axonal neuropathy

| **Study** | **Sample size (number of male patients)** | **Age**  **(years)** | **GBS symptoms** | **GBS sub-type** | **Timing between Covid-19 symptoms onset and GBS onset (days)** | **Treatments received** | **CSF Examination** | **Electrophysiology** | **Outcome** |
| --- | --- | --- | --- | --- | --- | --- | --- | --- | --- |
| Padroni et al. (2020) | 1 (0) | 70 | Medical Research Council grade 4/5, symmetric distal upper and lower limbs weakness, asthenia, hands and feet paresthesia, absent DTR, preserved light touch and pinprick sensation and gait difficulties | ASAMP | Covid-19 preceded GBS by 23 days | 400 mg/dL lintravenous immunoglobulin (IVIg) for 5 days,  Intubation and mechanical ventilation | Slight albumino-  cytological dissociation (CSF proteins = 48 mg/dL,  normal = 0–40 mg/dL, white blood cells = 1 × 106/L, normal  = 0–8 × 106/L). | Delayed or not evocable distal latency,  decreased amplitude,  decreased or not evocable  conduction velocity and  absent F waves in distal extremities  nerves | Patient was intubated and placed in mechanical ventilation for respiratory failure due to worsening of muscle weakness 4 days after admission |

**Key**: DTR= deep tendon reflex, CSF=cerebrospinal fluid, ASAMP= Acute sensory and motor polyradicul-oneuritis

| **Study** | **Sample size (number of male patients)** | **Age**  **(years)** | **GBS symptoms** | **GBS sub-type** | **Timing between Covid-19 symptoms onset and GBS onset (days)** | **Treatments received** | **CSF Examination** | **Electrophysiology** | **Outcome** |
| --- | --- | --- | --- | --- | --- | --- | --- | --- | --- |
| Alberti et al. (2020) | 1 (1) | 71 | Symmetric limb  weakness (Medical Research Councils core 3/5 at upper limbs and 2/5 at lower limbs), symmetric and extensive stocking-and-glove hypesthesia at the 4 limbs (lower limbs>upper limbs)  severe paresthesia in both hands and feet, absent DTR and low grade low back pain | AMAN | GBS preceded Covid-19 by 10 days | High-dose IV immunoglobulins (0.4 g/kg/d for 5 days), high-flow 60%–80% oxygen via nonrebreather mask, antiviral therapy (lopinavir + ritonavir), and hydroxychloroquine. | Mild increase in the protein content (54 mg/dL) and mild leukocytosis (9 cells/μL); CSF was negative efor SARS-CoV-2 | Absence of both the sural nerve SAP, increased tibial nerve CMAP,  delayed CMAP, distal latency, decreased velocity, decreased CMAP amplitude in peroneal  nerve,  decreased ulnar SAP and  absent F waves in distal extremities nerves. | Patient died within 24 hours due to progressive respiratory failure that was not responsive to continuous positive airway pressure ventilation  and prone positioning. |

**Key**: DTR= deep tendon reflex, CSF=cerebrospinal fluid, AMAN= Acute motor axonal neuropathy, CMAP= compound muscle action potential, SAP=sensory nerve action potential

| **Study** | **Sample size (number of male patients)** | **Age**  **(years)** | **GBS symptoms** | **GBS sub-type** | **Timing between Covid-19 symptoms onset and GBS onset (days)** | **Treatments received** | **CSF Examination** | **Electrophysiology** | **Outcome** |
| --- | --- | --- | --- | --- | --- | --- | --- | --- | --- |
| Virani et al. (2020) | 1 (1) | 54 | Weakness of lower extremities (2/5 strength in his lower extremities with 3/5 in his upper  extremities), numbness of lower extremities and absent DTR | AMAN | Covid-19 preceded GBS by 10 days | 400 mg/kg of intravenous immuneglobulin (IVIG) therapy for a planned 5-day course. Mechanical ventilation hydroxychloroquine 400 mg for the first two doses with subsequent 200 mg dose twice a day for an additional eight doses, and Physiotherapy | Nil | Nil | Patient was transitioned out of the ICU to a rehabilitation facility where he continued with physiotherapy |

**Key**: DTR= deep tendon reflex, CSF=cerebrospinal fluid, AMAN= Acute motor axonal neuropathy,

| **Study** | **Sample size (number of male patients)** | **Age**  **(years)** | **GBS symptoms** | **GBS sub-type** | **Timing between Covid-19 symptoms onset and GBS onset (days)** | **Treatments received** | **CSF Examination** | **Electrophysiology** | **Outcome** |
| --- | --- | --- | --- | --- | --- | --- | --- | --- | --- |
| Zhao et al. (2020) | 1 (0) | 61 | Acute weakness in both lower limbs and severe fatigue (strength; 4/5 in both arms and hands and 3/5 in both legs and feet), distally decreased sensation of light touch and pinprick and absent DTR. | DN | GBS preceded Covid-19 by 7 days | Nil | CSF: normal cell counts (5 × 10^6^/L, normal: 0–8 × 10^6^/L) and increased protein level (124 mg/dL, normal: 8–43 mg/dL) | Delayed  distal latencies and  absent F waves in distal extremities  nerves | At discharge on  day 30, she had normal muscle strength  in both arms and legs and return of  tendon reflexes in both legs and feet.  Her respiratory symptoms resolved  as well. |

**Key**: DTR= deep tendon reflex, CSF=cerebrospinal fluid, DN= Demyelinat-ing neuropathy,

| **Study** | **Sample size (number of male patients)** | **Age**  **(years)** | **GBS symptoms** | **GBS sub-type** | **Timing between Covid-19 symptoms onset and GBS onset (days)** | **Treatments received** | **CSF Examination** | **Electrophysiology** | **Outcome** |
| --- | --- | --- | --- | --- | --- | --- | --- | --- | --- |
| Toscano et al. (2020) | 5 (4) | Case 1: 77  Case 2: 23  Case 3: 55  Case 4: 76  Case 5: 61 | Case1: Flaccid areflexic tetraplegia,  Facial weakness, upper-limb paresthesia, paresthesia in the lower limbs and hands, bulbar symptoms (dysphagia, tongue weakness)  Case 2: Facial dyplegia,general areflexia, limb paresthesia with ataxia, fever and pharyngitis  Case 3:Flaccid tetraparesis,  facial weakness  areflexia, neck pain, paresthesia in the four limbs and lower limb weakness, fever, and cough | Case 1: SMAN  Case 2: SMAN  Case3: AMAN  Case 4: DN  Case 5: DN | Covid-19 preceded GBS by 5 to 10 days | Case 1: IVIG  Case 2: Amoxicillin  IVIG+ICU admission (non-invasivemechanicalventilation)  Case 3: IVIG+ICU admission  Case 4: IVIG  Case 5: IVIG+ICU admission (intubation-mechanical ventilation | Abnormal protein level and  white-cell count at day 10 in case 1 and in day 3 in cases 3 and 5 | Delayeddistallatency  Decreased amplitude Decreasedorconductionvelocity,  Absent F waves in distal extremities  Nerves for all patients | At 4 weeks after treatment, two patients  remained in the intensive, two were undergoing  physical therapy because of flaccid  paraplegia and had minimal upper-limb movement,  and a patient who was able to walk independently was discharged |

**Key**: DTR= deep tendon reflex, CSF=cerebrospinal fluid, SMAN= Sensory-motor axonal neuropathy, AMAN= Acute motor axonal neuropathy, DN= Demyelinating neuropathy

| **Study** | **Sample size (number of male patients)** | **Age**  **(years)** | **GBS symptoms** | **GBS sub-type** | **Timing between Covid-19 symptoms onset and GBS onset (days)** | **Treatments received** | **CSF Examination** | **Electrophysiology** | **Outcome** |
| --- | --- | --- | --- | --- | --- | --- | --- | --- | --- |
| Toscano et al. (2020) |  |  | Case 4: Flaccid areflexic tetrapresis  ataxia, lumber pain, cough, hiposmia  Case 5: Flaccid areflexic, paralegia, difficulties in climbing stairs, lower limb paresthesia, cough,  ageusia and anosmia |  |  |  |  |  |  |

**Key**: DTR= deep tendon reflex, CSF=cerebrospinal fluid

| **Study** | **Sample size (number of male patients)** | **Age**  **(years)** | **GBS symptoms** | **GBS sub-type** | **Timing between Covid-19 symptoms onset and GBS onset (days)** | **Treatments received** | **CSF Examination** | **Electrophysiology** | **Outcome** |
| --- | --- | --- | --- | --- | --- | --- | --- | --- | --- |
| Riva et al. (2020) | 1 (1) | 60 | Progressive weakness,  distal paresthesia at four-limbs, severe vibratory sensation and proprioception deficit at lower limbs and absent DTR | AIDP | Covid-19 preceded GBS by 20 days | IVIG (0.4 g/kg/d for 5 days) | Normal cell count and protein levels. | Reduced  conduction velocities, reduced SAP and  CMAP amplitudes with  sural nerve sparing and abnormal temporal dispersion of  peroneal nerves cMAP | Muscle weakness worsened and rapidly  spread distally and to thoracic and cranial nerves causing  facial diplegia, hypophonia and dysarthria. However, no ventilation or feeding tube support was  required. |

**Key**: DTR= deep tendon reflex, CSF=cerebrospinal fluid, AIDP= Acute inflammatory

demyelinating polyneuropathy

| **Study** | **Sample size (number of male patients)** | **Age**  **(years)** | **GBS symptoms** | **GBS sub-type** | **Timing between Covid-19 symptoms onset and GBS onset (days)** | **Treatments received** | **CSF Examination** | **Electrophysiology** | **Outcome** |
| --- | --- | --- | --- | --- | --- | --- | --- | --- | --- |
| Ottaviani et al. (2020) | 1 (0) | 66 | Rapidly progressive symmetric weakness in the lower limbs, initial distal weakness in the upper limbs (MRC 4/5),  diffuse areflexia and no sensory deficits | MPDAD | Covid-19 preceded GBS by 10 days | IVIG ( 0.4 g/kg for 5 days), antiretroviral drugs (Lopinavir and Ritonavir) and hydroxychloroquine | Normal cell count and protein levels. | Absence of F-waves, diffuse prolonged distal motor latencies, reduced distal CMAP amplitudes,  slight reduction of conduction velocities | Developed progressive weakness in all limbs, dysesthesia, and unilateral facial  palsy |

**Key**: DTR= deep tendon reflex, CSF=cerebrospinal fluid, MPDAD= mixed pattern of demyelination and axonal damage

| **Study** | **Sample size (number of male patients)** | **Age**  **(years)** | **GBS symptoms** | **GBS sub-type** | **Timing between Covid-19 symptoms onset and GBS onset (days)** | **Treatments received** | **CSF Examination** | **Electrophysiology** | **Outcome** |
| --- | --- | --- | --- | --- | --- | --- | --- | --- | --- |
| Rana et al. (2020) | 1 (1) | 54 | Quadriparesis, weak in his lower extremities (MRC 0–1/5 in all muscles) and 3/5 in proximal and 4/5 in distal muscles of the upper extremities, facial diplegia  areflexia, burning dysesthesias,  opthalmoparesis, dysautonomia | DP | Covid-19 preceded GBS by 14 days | A 5-day regimen of IVIG (400mg/kg daily), intubation (4 day),  hydroxychloroquine and azithromycin | Nil | Prolonged distal latencies, conductions blocks, and slowing of conduction velocities,  F and H waves were absent as were all sensory responses, except the sural | Patient was discharged to inpatient rehabilitation facility after 7 days on admission |

**Key**: DTR= deep tendon reflex, CSF=cerebrospinal fluid, DP= Demyelinating polyneuropathy

| **Study** | **Sample size (number of male patients)** | **Age**  **(years)** | **GBS symptoms** | **GBS sub-type** | **Timing between Covid-19 symptoms onset and GBS onset (days)** | **Treatments received** | **CSF Examination** | **Electrophysiology** | **Outcome** |
| --- | --- | --- | --- | --- | --- | --- | --- | --- | --- |
| Caamano & Beato et al. (2020) | 1 (1) | 61 | Facial diplegia | MFS | Covid-19 preceded GBS by 14 days | Hydroxychloroquineand Lopinavir/Ritonavir for 14 days,  Low dose oral prednisone | Mildly elevated levels of proteins(44 mg/dL), absent leukocytes and a negative RT-PCR for SARS-CoV-2 on CSF | Nil | Barely notable improvement was observed in both sides of the face |

**Key**: DTR= deep tendon reflex, CSF=cerebrospinal fluid, MFS= Miller Fisher syndrome

| **Study** | **Sample size (number of male patients)** | **Age**  **(years)** | **GBS symptoms** | **GBS sub-type** | **Timing between Covid-19 symptoms onset and GBS onset (days)** | **Treatments received** | **CSF Examination** | **Electrophysiology** | **Outcome** |
| --- | --- | --- | --- | --- | --- | --- | --- | --- | --- |
| Gutiérrez-Ortiz et al. (2020) | 2 (2) | Case 1:50  Case 2: 39 | Case 1: vertical diplopia, perioral paraesthesias,  Gait instability,  a broad-based ataxic gait, absent DTR in the upper and in the lower limbs.  Case 2: bilateral abducens palsy, all DTR were absent | MFS | Case 1: Covid-19 preceded GBS by 5 days  Case 2: Covid-19 preceded GBS by 3 days | Case 1: IVIG (0.4 g/kg for 5 days  Case 2: acetaminophen | Case 1: opening pressure of 11 cm of H2O.  Case 2: opening pressure of 10 cm of H2O.  Cases 1 and 2: raised white blood cell count, protein and glucose levels with normal cytology, sterile cultures and negative serologies, including the rRT-PCR for COVID-19 | Nil | Case 1: ataxia improved and the patient was discharged home two weeks after admission  Case 2: Patient was discharged home and treated via telemedicine due to hospital saturation |

**Key**: DTR= deep tendon reflex, CSF=cerebrospinal fluid, MFS= Miller Fisher syndrome

| **Study** | **Sample size (number of male patients)** | **Age**  **(years)** | **GBS symptoms** | **GBS sub-type** | **Timing between Covid-19 symptoms onset and GBS onset (days)** | **Treatments received** | **CSF Examination** | **Electrophysiology** | **Outcome** |
| --- | --- | --- | --- | --- | --- | --- | --- | --- | --- |
| Camdessa-ncheet al. (2020) | 1 (1) | 60 | Paraesthesis in feet and hands. Muscle strength: 2/5 in the legs, 2/5 the arms, 3/5 in the forearms and 4/5 in the hands. Areflexia in the four limbs.  Flaccid severe tetraparesia.  Swallowing disturbance. | DP | Covid-19 preceded GBS by 11 days | An intravenous immunoglobulin treatment (0.4 g/kg per day during 5 days). | Protein level was 1.66 g per liter and cell count normal. | Delayed or not evocable distal latency, decreased amplitude, decreased or not evocable conduction velocity and block and absent F waves in distal extremities nerves | Not reported |

**DP=demylinating polyneuropathy**

| **Study** | **Sample size (number of male patients)** | **Age**  **(years)** | **GBS symptoms** | **GBS sub-type** | **Timing between Covid-19 symptoms onset and GBS onset (days)** | **Treatments received** | **CSF Examination** | **Electrophysiology** | **Outcome** |
| --- | --- | --- | --- | --- | --- | --- | --- | --- | --- |
| Diez-Porras et al. (2020) | 1 (1) | 54 | Hypoesthesia in the left mandibular region and distal upper limb, paraparesis of upper limbs and difficulty walking. Areflexia. Muscle strength: 2/5 left and 3/5 upper limbs. Bilateral facial diplagia and dysphagia | ADP | Covid-19 preceded GBS by 5 days | Azithromycin, hydroxychloroquine, lopinavir/ritonavir, intravenous immunoglobulins (IVIg) at 0.4 g/kg/day for 5 days, invasive ventilation and  rehabilitation | mild albuminocytologic dissociation (protein levels 52 mg/dL and absence of leukocytes). | conduction blocks, absence of F waves in the right ulnar nerve and axon potentials in the F response of the right tibial nerve of diffuse distribution, but mainly affecting the nerves of the upper limbs | Responded very well to rehabilitation and was able to walk independently at discharge time |

**ADP= Acute demylinating polyneuropathy**

| **Study** | **Sample size (number of male patients)** | **Age**  **(years)** | **GBS symptoms** | **GBS sub-type** | **Timing between Covid-19 symptoms onset and GBS onset (days)** | **Treatments received** | **CSF Examination** | **Electrophysiology** | **Outcome** |
| --- | --- | --- | --- | --- | --- | --- | --- | --- | --- |
| El Outmani al. (2020) | 1 (0) | 70 | Quadriplegia, hypotonia,  areflexia and bilateral positive Lasegue sign | Acute Motor and Sensory Axonal Neuropathy (AMSAN) | Covid-19 preceded GBS by 3 days | intravenous  immunoglobulin (2 g/kg for 5 days) and a combination of  Hydroxychloroquine (600 mg per day) and Azithromycine  (500 mg at the first day, then 250 mg per day) | increased protein level at 1 g per liter (normal range: 0,2–0,4)  with normal white blood cell count.. | marked  reduction or absence of electrical potentials in both motor and  sensory nerves in all four limbs, with little or no abnormalities  in conduction velocities and latencies. | No significant  neurological improvement was seen after one week of treatment |

| **Study** | **Sample size (number of male patients)** | **Age**  **(years)** | **GBS symptoms** | **GBS sub-type** | **Timing between Covid-19 symptoms onset and GBS onset (days)** | **Treatments received** | **CSF Examination** | **Electrophysiology** | **Outcome** |
| --- | --- | --- | --- | --- | --- | --- | --- | --- | --- |
| Zito et al. (2020) | 1 (1) | 57 | numbness and tingling in the feet  and hands. weakness in the dorsiflexion of the foot  and the extension of the toes,  gait ataxia, loss of touch and vibration sensation in the feet and  ankles reduced or absent reflexes | Acute Motor and Sensory Axonal Neuropathy (AMSAN) | Covid-19 preceded GBS by 12 days | An intravenous immunoglobulin (IVIG) cycle at 0.4 g/kg/day over 5 days. | normal cell count.proteins, CSF/serum albumin ratio, and absence of  oligoclonal banding. | reduced or absent  compound muscle action potentials and sensory nerve action  potentials in the lower limbs, absent F wave response in the  lower limbs, and prolonged F wave response in the upper  limbs. | He slowly improved through physiotherapy and, after 1  month, he was able to walk without aid and was discharged |

| **Study** | **Sample size (number of male patients)** | **Age**  **(years)** | **GBS symptoms** | **GBS sub-type** | **Timing between Covid-19 symptoms onset and GBS onset (days)** | **Treatments received** | **CSF Examination** | **Electrophysiology** | **Outcome** |
| --- | --- | --- | --- | --- | --- | --- | --- | --- | --- |
| Assini al. (2020) | 2 (2) | Case 1: 55  Case 2: 60 | Case 1: bilateral eyelid ptosis, dysphagia, and dysphonia.  hyporeflexia of upper and lower limbs, without  muscle weakness.  Case 2: acute weakness in lower limbs, gastroplegia, paralytic ileus, and loss of  blood pressure control . Areflexia. | Case 1: Miller Fisher syndrome  Case 2: Sensory-motor axonal polyneuropathy | Cases 1 and 2: Covid-19 preceded GBS by 20 days | Case 1: idrossichlorochine,  Arbidol, ritonavir, and Lopinavir,  invasive ventilation, and intravenous immunoglobulins  Case 2: intravenous  immunoglobulin therapy was started at the standard  dosage of 0.4 g/kg/day | Case 1: presence of oligoclonal bands both in CSF and serum, with increased IgG/albumin ratio in CSF (233); total protein level  Was normal  Case 2: presence of oligoclonal bands both in CSF with increased ratio IgG/albumin in CSF (170); total protein  level in CSF was normal. | Case 1: a symmetric demyelinating findings and  a typical sural sparing pattern.  Case 2: The amplitudes of sensory and  motor action potentials were significantly reduced. | Case 1: Progressive and complete symptoms remission  Case 2: symptoms remarkably improved. |

| **Study** | **Sample size (number of male patients)** | **Age**  **(years)** | **GBS symptoms** | **GBS sub-type** | **Timing between Covid-19 symptoms onset and GBS onset (days)** | **Treatments received** | **CSF Examination** | **Electrophysiology** | **Outcome** |
| --- | --- | --- | --- | --- | --- | --- | --- | --- | --- |
| Klinc et al. (2020) | 1 (1) | 50 | Facial diplegia, mild symmetric proximal  muscle weakness and impaired proprioception in the  legs, ataxic gait and areflexia. | sensorimotor, predominantly  demyelinating polyradiculoneuropathy | Covid-19 preceded GBS by 28 days | intravenous Ig (2 g/kg in 5 days) | normal cell count and total  protein level | Signs of polyradiculoneuropathy | Patient progresively recoverd and was discharged |
| Oguz-Akarsu et al. (2020) | 1 (0) | 53 | Mild dysarthria, bilateral upper and lower limb weakness, areflexia in the lower limbs, and reduced sensation to pin prick. She could walk only with assistance. | Demyelinating polyradiculoneuropathy) | Indeterminate- it was not reported | Hydroxychloroquine and azithromycin. | A protein level of  32.6 mg/dL with no leucocytes | Nil | Neurological symptoms improved remarkably after 2 weeks. |

| **Study** | **Sample size (number of male patients)** | **Age**  **(years)** | **GBS symptoms** | **GBS sub-type** | **Timing between Covid-19 symptoms onset and GBS onset (days)** | **Treatments received** | **CSF Examination** | **Electrophysiology** | **Outcome** |
| --- | --- | --- | --- | --- | --- | --- | --- | --- | --- |
| Chan et al. (2020) | 1 (1) | 58 | Acute-onset bilateral facial weakness,  dysarthria, and paresthesia in his feet, areflexia in the lower extremities | Acute inflammatory demyelinating  polyneuropathy. | Indeterminate | Ceftriaxone, Azithromycin and IVIG at 0.4 g/kg/day | Cytoalbuminologic dissociation with a  protein of 1.00 g/L (normal 0.15–0.45 g/L) and a white blood  cell count of 4 × 106/L (normal ≤ 5 × 106/L) | Absent  blink reflexes bilaterally and an absent F wave in the left  tibial nerve | Discharged with no much symptoms improvement |

| **Study** | **Sample size (number of male patients)** | **Age**  **(years)** | **GBS symptoms** | **GBS sub-type** | **Timing between Covid-19 symptoms onset and GBS onset (days)** | **Treatments received** | **CSF Examination** | **Electrophysiology** | **Outcome** |
| --- | --- | --- | --- | --- | --- | --- | --- | --- | --- |
| Bigaut et al. (2020) | Case 1: 1 (1)  Case 2: 1 (0) | Case 1:43  Case 2: 70 | Case 1:a rapidly progressive paraesthesia, hypoesthesia, and distal weakness in the lower limbs, ataxia and a right peripheral facial palsy and areflexia in the lower limb  Case 2: Flaccid tetraparesis,  generalized areflexia,  forelimb paresthesia | Case 1: Acute inflammatory demyelinating  Polyneuropathy  Case 2:acute inflammatory demyelinating  polyneuropathy | Case 1: GBS preceded Covid-19 by 7 days  Case 2: Covid-19 preceded GBS by 7 days | Case 1: IV immunoglobulin infusions.  Case 2: IVIg  (2 g/kg); | Case 1: normal cell count (1 × 106/L) and increased protein level (0.94 g/L)  Case 2: protein  level: 1.6 g/L; cell  count: 6 × 106/L | Case 1: Conduction blocks and decease conduction velocity in both peroneal and tibial nerves, a sural sparing pattern, abolition of the H-reflex, and increased F-wave latencies.  Case 2: motor and  sensitive decreased  conduction velocities  and conduction blocks | Case 1: Progressively improved and was discharged  Case 2: slow  progressive  improvement |

| **Study** | **Sample size (number of male patients)** | **Age**  **(years)** | **GBS symptoms** | **GBS sub-type** | **Timing between Covid-19 symptoms onset and GBS onset (days)** | **Treatments received** | **CSF Examination** | **Electrophysiology** | **Outcome** |
| --- | --- | --- | --- | --- | --- | --- | --- | --- | --- |
| R eyes-Bueno, al. (2020) | 1 (0) | 51 | Paresis of the left external rectus muscle with horizontal  diplopia, discrete bilateral facial paresis, lower limb weakness and global areflexia | Miller-  Fisher Syndrome | Covid-19 preceded GBS by 15 days | IVIG 0.4mg/Kg/24h for 5 days and  gabapentin 900 mg/24h | high protein levels with albumin-cytological dissociation  (70mg/dl of proteins and 5 leukocytes). | F-wave latency for the lower limbs and low A-wave amplitude on the left leg. Poor muscle activity on EMG | Progressive improvement in facial and limb paresis,  diplopia and pain. She is still on neurological rehabilitation |

| **Study** | **Sample size (number of male patients)** | **Age**  **(years)** | **GBS symptoms** | **GBS sub-type** | **Timing between Covid-19 symptoms onset and GBS onset (days)** | **Treatments received** | **CSF Examination** | **Electrophysiology** | **Outcome** |
| --- | --- | --- | --- | --- | --- | --- | --- | --- | --- |
| Marta-Enguita et al. (2020) | 1 (0) | 76 | lower back pain radiating to the backs  of the legs and progressive tetraparesis with distal-onset  paraesthesia. Pain was bilateral, predominantly affecting  the right side; it was more intense during the night, leading  to difficulties falling asleep.  Dysphagia | Acute inflammatory demyelinating  Polyneuropathy | Covid-19 preceded GBS by 8 days | Nonsteroidal anti-inflammatory drugs, pyrazolones, and  transdermal morphine.  amoxicillin/ clavulanic acid and azithromycin | Nil | .Nil | The patient finally died 12 hours after onset of the bulbar  symptoms |

| **Study** | **Sample size (number of male patients)** | **Age**  **(years)** | **GBS symptoms** | **GBS sub-type** | **Timing between Covid-19 symptoms onset and GBS onset (days)** | **Treatments received** | **CSF Examination** | **Electrophysiology** | **Outcome** |
| --- | --- | --- | --- | --- | --- | --- | --- | --- | --- |
| Helbok et al. (2020) | 1 (1) | 68 | Decreased sensation to touch and pinprick in the lower  extremities, absent ankle jerk, atactic stance and  inability to walk without assistance. | Acute inflammatory demyelinating  Polyneuropathy | Indeterminate | Intravenous  immunoglobulin therapy (IVIG, 30 g) was initiated.  Supplemental oxygen and ventilation due to worsening of respiratory symptom. Rehabilitation | Normal cell counts  (2/mm [3]) and protein level (64 mg/dl) and a serum/  CSF glucose ratio of 0.83.  Anti-SARSCoV-  2- antibodies were highly positive in serum and  CSF | F-wave abnormalities in all  nerves, delayed distal motor latency in one nerve,  reduced distal amplitudes in two and a sural-sparing  pattern | The patient improved gradually  and regained  mobility without significant help. |

| **Study** | **Sample size (number of male patients)** | **Age**  **(years)** | **GBS symptoms** | **GBS sub-type** | **Timing between Covid-19 symptoms onset and GBS onset (days)** | **Treatments received** | **CSF Examination** | **Electrophysiology** | **Outcome** |
| --- | --- | --- | --- | --- | --- | --- | --- | --- | --- |
| Coen et al. (2020) | 1 (1) | 70s | Distal allodynia, difficulties in voiding and constipation. Bilateral lower limb  flaccid paresis, absent deep tendon reflexes of the upper and lower limb | Acute inflammatory demyelinating  polyneuropathy | Covid-19 preceded GBS by 10 days | Intravenous  immunoglobulins (IVIg; IgPro10, Privigen®; 0.4 g/kg/day for 5 days) | Albuminocytologic dissociation  without intrathecal IgG synthesis | Sural sparing pattern and decreased  persistence or absent F-waves in tested nerves | Improvement was rapid and patient was referred for Neurorehabilitation. |

| **Study** | **Sample size (number of male patients)** | **Age**  **(years)** | **GBS symptoms** | **GBS sub-type** | **Timing between Covid-19 symptoms onset and GBS onset (days)** | **Treatments received** | **CSF Examination** | **Electrophysiology** | **Outcome** |
| --- | --- | --- | --- | --- | --- | --- | --- | --- | --- |
| Su al. (2020) | 1 (1) | 72 | Symmetric paresthesias  and ascending appendicular weakness and absence of reflexes | Acute inflammatory demyelinating  polyneuropathy | Covid-19 preceded GBS by 6 days | Intravenous immunoglobulin  2 g/kg between days 3 and 6  sulfamethoxazole-trimethoprim.  tracheostomy  and percutaneous endoscopic gastrostomy tubes | WBC 1 cell/μL  and protein 313 mg/dL; | Diffusely  decreased velocities, conduction block, and absent F waves. | He remains in the  ICU with severe weakness |

| **Study** | **Sample size (number of male patients)** | **Age**  **(years)** | **GBS symptoms** | **GBS sub-type** | **Timing between Covid-19 symptoms onset and GBS onset (days)** | **Treatments received** | **CSF Examination** | **Electrophysiology** | **Outcome** |
| --- | --- | --- | --- | --- | --- | --- | --- | --- | --- |
| Arnaud et al. (2020) | 1 (1) | 64 | Areflexia, severe  flaccid paraparesis, mainly affecting proximal muscles, and a  decreased proprioceptive length-dependent sensitivity involving  the four limbs, hypoesthesia to light touch and pinprick  in lower extremities | Acute inflammatory demyelinating  polyneuropathy | Covid-19 preceded GBS by 16 days | Cefotaxime, Azithromycin and Hydroxychloroquine.  intravenous immunoglobulin for five days | Increased protein level at  1.65 g/L, no pleocytosis and no intrathecal synthesis of  immunoglobulins. PCR assays of the CSF were negative for SARSCoV-  2 | Delayed  motor distal latencies in the upper limbs, absence of F-waves in the 4 limbs, conduction blocks in peroneal and tibial nerves bilaterally,  very low motor conduction velocities, and absent sensory  nerve action potentials except for radial nerves and median nerves  at the palm | Not reported |

| **Study** | **Sample size (number of male patients)** | **Age**  **(years)** | **GBS symptoms** | **GBS sub-type** | **Timing between Covid-19 symptoms onset and GBS onset (days)** | **Treatments received** | **CSF Examination** | **Electrophysiology** | **Outcome** |
| --- | --- | --- | --- | --- | --- | --- | --- | --- | --- |
| Lantos et al. (2020) | 1 (1) | 36 | Progressive ophthalmoparesis  (including initial left CN III and eventual bilateral  CN VI palsies), ataxia, and hyporeflexia, paresthesia in both legs | Miller Fisher syndrome | Covid-19 preceded GBS by 4 days | Intravenous immunoglobulin and hydroxychloroquine | Nil | Nil | patient was discharged after 4 days of hospitalization |

| **Study** | **Sample size (number of male patients)** | **Age**  **(years)** | **GBS symptoms** | **GBS sub-type** | **Timing between Covid-19 symptoms onset and GBS onset (days)** | **Treatments received** | **CSF Examination** | **Electrophysiology** | **Outcome** |
| --- | --- | --- | --- | --- | --- | --- | --- | --- | --- |
| Valayos Galan et al. (2020) | 1 (1) | 43 | Symmetrical weakness involving all 4 limbs, inability to walk, sensory alterations in distal regions of  all 4 limbs and bilateral facial palsy and dysphagia | Demyelinating polyradiculoneuropathy | Covid-19 preceded GBS by 10 days | Hydroxychloroquine sulfate, antiretroviral drugs (lopinavir and ritonavir), antibiotics  (amoxicillin), corticosteroids, and low-flow oxygen therapy | Nil | Increased  distal motor latency and decreased sensory nerve conduction  velocity in the nerves evaluated, and increased minimal  F-wave latency in the right L5 and S1 spinal nerve roots | Subsequently, neurological and respiratory symptoms  progressed favourably |

| **Study** | **Sample size (number of male patients)** | **Age**  **(years)** | **GBS symptoms** | **GBS sub-type** | **Timing between Covid-19 symptoms onset and GBS onset (days)** | **Treatments received** | **CSF Examination** | **Electrophysiology** | **Outcome** |
| --- | --- | --- | --- | --- | --- | --- | --- | --- | --- |
| Webb et al. (2020) | 1 (1) | 57 | Progressive limb weakness  and foot dysaesthesia. Difficulty  standing unaided and noticed some tingling sensations  in his feet and unable to stand, and his arms felt weak.  impaired vibration sense to the  hips bilaterally. | Acute  inflammatory demyelinating polyneuropathy | Covid-19 preceded GBS by 7days | Intravenous immunoglobulin (IVIG) 2 g/kg divided over 5 days  was started 24 hours after presentation to the hospitaly.  Mechanical ventilation | high cerebrospinal fluid (CSF)  protein (0.51 g/L) with normal glucose and cell counts. No  organisms were found on gram staining and viral PCR, including  SARS-CoV-  2 RNA, was negative. | Reduced conduction  velocity and prolonged distal motor latencies in motor and  sensory nerves in the upper and lower limbs. Prolonged F-wave latency in the right  ulnar nerve. Reduced  velocities in both superficial peroneal and right sural nerves. | Symptoms initially deteriorated, but improved after sometime and the patient was weaned off ventilator |

| **Study** | **Sample size (number of male patients)** | **Age**  **(years)** | **GBS symptoms** | **GBS sub-type** | **Timing between Covid-19 symptoms onset and GBS onset (days)** | **Treatments received** | **CSF Examination** | **Electrophysiology** | **Outcome** |
| --- | --- | --- | --- | --- | --- | --- | --- | --- | --- |
| Loscano et al. (2020) | Case 1: 1 (0)  Case 2:  1 (0) | Case 1: 52  Case 2: 63 | Case 1: back pain, distal weakness, paraesthesia, dysgeusia, cacosmia, tetraplegia, areflexia and respiratory failure, dysautonomia  Case 2: lower limb pain, mild weakness, distal paraesthesia, tetraparesis and areflexia | Cases 1, 2 and 3:  acute inflammatory demyelinating  polyneuropathy | Case 1: Covid-19 preceded GBS by 15 days  Case 2: Covid-19 preceded GBS by 7 days | Case 1, 2 and 3: 1 cycle of intravenous immunoglobulin | Case 1: White blood cell count 3 cells/  μL; protein level 60mg/dl; negative PCR assay for SARS-COV-2  Case 2: White blood cell count 2 cells/  μL; protein level 40mg/d | Increased  distal motor latency and decreased sensory nerve conduction  velocity and increased minimal  F-wave latency in the right L5 and S1 spinal nerve roots | Case 1: Able to stand with assistance. GBS clinical disability score=4/6  Case 2: GBS clinical disability score=1/6 |

| **Study** | **Sample size (number of male patients)** | **Age**  **(years)** | **GBS symptoms** | **GBS sub-type** | **Timing between Covid-19 symptoms onset and GBS onset (days)** | **Treatments received** | **CSF Examination** | **Electrophysiology** | **Outcome** |
| --- | --- | --- | --- | --- | --- | --- | --- | --- | --- |
| Loscano et al. (2020)  (continued) | Case 3:  1 (0) | Case 3: 61 | Case 3: lower limb weakness, distal paraesthesia, dysautonmia, dizziness, dysphagia, worsening bulbar symptoms and facial nerve palsy and areflexia |  | Case 3: Covid-19 preceded GBS by 22 days | Intravenous immunoglobulin (IVIG) 2 g/kg divided over 5 days  was started 24 hours after presentation to the hospitaly.  Mechanical ventilation | Case 3: White blood cell count 4 cells/  μL; protein level 140mg/d;  negative PCR assay for SARS-COV-2 |  | Case 3: walk with assistance.  Persistent paraesthesia and neuropathicpain GBS clinical disability score=3/6 |

| **Study** | **Sample size (number of male patients)** | **Age**  **(years)** | **GBS symptoms** | **GBS sub-type** | **Timing between Covid-19 symptoms onset and GBS onset (days)** | **Treatments received** | **CSF Examination** | **Electrophysiology** | **Outcome** |
| --- | --- | --- | --- | --- | --- | --- | --- | --- | --- |
| Scheidl et al. (2020) | 1 (0) | 54 | Proximally pronounced, moderate, symmetric paraparesis in the lower extremities. Areflexia, numbness, and tingling of all  extremities. | Acute inflammatory demyelinating  polyneuropathy | Covid-19 preceded GBS by 21 days | Intravenous immunoglobulin at the dose of 0.4 g/kg/day | Albuminocytologic dissociation with increased protein level  (140 g/L) and normal cell count, immunoassay, and Lyme-serology were  negative. SARS-Cov-2 RNA was not tested in CSF | Significantly prolonged distal motor latencies and temporal dispersion  of the CMAP of the common peroneal nerve bilaterally. Normal F-wave latencies  with pathological intermediate latency responses (complex A-waves) on  both sides . | Almost complete  recovery |

| **Study** | **Sample size (number of male patients)** | **Age**  **(years)** | **GBS symptoms** | **GBS sub-type** | **Timing between Covid-19 symptoms onset and GBS onset (days)** | **Treatments received** | **CSF Examination** | **Electrophysiology** | **Outcome** |
| --- | --- | --- | --- | --- | --- | --- | --- | --- | --- |
| Manji et al. (2020) | 1 (1) | 12 | Progressive symmetric ascending quadriparesis with bilateral facial paresis. Lost function of both lower limbs with associated weakness of bilateral upper limbs as well and could not get out of bed. Areflexia | Demyelinating polyradiculoneuropathy | Covid-19 preceded GBS by 7 days | Cardio-Pulmonary Resuscitation, intubation and ventilation and positive inotrope infusion.  400mg/kg of intravenous immune globulin [IVIG]. Physiotherapy | Nil | Nil | Initially showed significant in muscle strength, but died eventually of cardiac arrest |

| **Study** | **Sample size (number of male patients)** | **Age**  **(years)** | **GBS symptoms** | **GBS sub-type** | **Timing between Covid-19 symptoms onset and GBS onset (days)** | **Treatments received** | **CSF Examination** | **Electrophysiology** | **Outcome** |
| --- | --- | --- | --- | --- | --- | --- | --- | --- | --- |
| Frank et al. (2020) | 1 (1) | 15 | Weakness and pain in the lower limbs, which ascended to his upper  limbs | Acute motor axonal neuropathy | Covid-19 preceded GBS by unspecified days | 400 mg/kg/day of intravenous immune  globulin (IVIG) therapy for a planned 5 days course.  Physiotherpy | No abnormalities. CSF negative for SARS COV-2 | Normal sensory  nerve action potential, though severe reduction  of the nerve compound muscle action potential amplitude  in all motor nerves studied, with relatively preserved  conduction velocities. F waves were absent in  the studied nerves. | Weakness in the upper and lower extremities persist, but undergoing physiotherapy at the moment |

| **Study** | **Sample size (number of male patients)** | **Age**  **(years)** | **GBS symptoms** | **GBS sub-type** | **Timing between Covid-19 symptoms onset and GBS onset (days)** | **Treatments received** | **CSF Examination** | **Electrophysiology** | **Outcome** |
| --- | --- | --- | --- | --- | --- | --- | --- | --- | --- |
| Khalifa et al. (2020) | 1 (1) | 11 | Unsteady  Gait, inability to walk or climb stairs, tingling sensation in both the legs and feet. Hypotonia, lost ankle and knee reflexes, impaired sensation regarding  pain and light touch of both feet up to the mid-legs with impaired  proprioception | Demyelinating polyneuropathy | Covid-19 preceded GBS by 41 days | Intravenous immunoglobulin, acetaminophen, hydroxychloroquine, thromboprophylaxis, and low-molecular-weight  heparin (R/enoxaparin) | Total cell count was 5 cells/μL, with  91% lymphocytes and 9% monocytes; CSF chemistry showed  chloride 116 mmol/L (normal range, 120–130 mmol/L), glucose  65 mg/dL (normal range, 40–70 mg/dL), and high protein  level (316.7 mg/dL [normal range, 15–45 mg/d | delayed latencies and  low amplitude with dispersion of compound muscle action potentials, and with no F-wave response with impaired sensory  conduction | Symptoms improved |

| **Study** | **Sample size (number of male patients)** | **Age**  **(years)** | **GBS symptoms** | **GBS sub-type** | **Timing between Covid-19 symptoms onset and GBS onset (days)** | **Treatments received** | **CSF Examination** | **Electrophysiology** | **Outcome** |
| --- | --- | --- | --- | --- | --- | --- | --- | --- | --- |
| Hutchins et al. (2020) | 1 (1) | 21 | Right-sided facial numbness and weakness. Dysarthria,  bilateral lower  extremity weakness, bilateral upper  extremity paresthesia, and continued  facial weakness, diffuse areflexia | Acute  sensorimotor polyneuropathy | Covid-19 preceded GBS by 16 days | 5 cycles of plasma exchange | The CSF was negative for  bacterial culture and stain and demonstrated  normal glucose levels (65 mg/dL),  no leukocytes, and mildly elevated protein  levels (49mg/dL). | Electromyography and nerve conduction  studies demonstrated findings consistent with an acute  sensorimotor polyneuropathy with both demyelinating and axonal  features | The patient was placed on rehabilitation |

| **Study** | **Sample size (number of male patients)** | **Age**  **(years)** | **GBS symptoms** | **GBS sub-type** | **Timing between Covid-19 symptoms onset and GBS onset (days)** | **Treatments received** | **CSF Examination** | **Electrophysiology** | **Outcome** |
| --- | --- | --- | --- | --- | --- | --- | --- | --- | --- |
| Bracaglia et al. (2020) | 1 (0) | 66 | Hyposthenia in all limbs, severe in lower, distal tingling sensation and pronounced lumbar pain for  about eight days. She was unable to walk, reported difficulty  in swallowing and speeching, tendon reflexes were abolished. Facial diplegia | Acute demyelinating polyneuritis | Indeterminate | Intravenous immune globulin (IvIg), ritonavir  100 mg and darunavir 800 mg per day with hydroxychloroquine  200 mg twice daily.  Rehabilitation | protein content 245 mg/dL, cells  13/mmc, polymorphonucleate 61.5% | Distal compound muscle action potentials (cMAP) showed reduced amplitude because of temporal dispersion. | Regained some muscle strength and was referred for rehabilitation |

| **Study** | **Sample size (number of male patients)** | **Age**  **(years)** | **GBS symptoms** | **GBS sub-type** | **Timing between Covid-19 symptoms onset and GBS onset (days)** | **Treatments received** | **CSF Examination** | **Electrophysiology** | **Outcome** |
| --- | --- | --- | --- | --- | --- | --- | --- | --- | --- |
| Lampe et al. (2020) | 1 (1) | 65 | Acute and progressive weakness of his right arm and  lower limbs, which caused recurrent falls.  Reduced reflexes | Demyelinating polyradiculoneuropathy | Covid-19 preceded GBS by 3 days | Intravenous immunoglobulin (IVIG) treatment  (0.4 g/kg bodyweight per day for 5 days.  Physiotherapy | slight increase  in protein level (56 mg/dl) with a normal cell count (2 cells/μl) | Prolonged distal motor latencies of the  right median and tibial nerves as well as increased F-wave  latencies of the median and tibial nerves on both sides | Significant improvement  in GBS symptoms. Hence, the patient was  discharged from the hospital 12 days after admission |

| **Study** | **Sample size (number of male patients)** | **Age**  **(years)** | **GBS symptoms** | **GBS sub-type** | **Timing between Covid-19 symptoms onset and GBS onset (days)** | **Treatments received** | **CSF Examination** | **Electrophysiology** | **Outcome** |
| --- | --- | --- | --- | --- | --- | --- | --- | --- | --- |
| Paybast et al. (2020) | Case 1: 1 (1)  Case 2: 1(0) | 38  14 | Case 1: symmetric progressive ascending quardiparesthesia. Bilateral facial droop, areflexia, decrease in sensation in 4 limbs.  Romberg test was positive.  Case 2: progressive ascending quadripareshtesia, mild lower limb weakness. Areflexia | Case 1: Acute demyelinating polyneuropathy  Case 2: acute demyelinating polyneuropathy | Case 1: Covid-19 preceded GBS by 21 days  Case 2: Indeterminate | Case 1: therapeutic plasma exchange  (TPE). labetalol intravenously  Case 2: intravenous immunoglobulin and orral hydroxychloroquine sulfate | Case 1: Normal and negative for SARS COV-2  Case 2: albuminocytologic dissociation | Case 1: considerable reduction in the  compound motor action potentials amplitude with prolonged distal  latency and reduced conduction velocity. F and H waves were absent.  Case 2: Nil | Case 1: Not reported  Case 2: improved and was discharged |

| **Study** | **Sample size (number of male patients)** | **Age**  **(years)** | **GBS symptoms** | **GBS sub-type** | **Timing between Covid-19 symptoms onset and GBS onset (days)** | **Treatments received** | **CSF Examination** | **Electrophysiology** | **Outcome** |
| --- | --- | --- | --- | --- | --- | --- | --- | --- | --- |
| Tiet & AlShaik (2020) | 1 (1) | 49 | Distal lower limb paraesthesia, difficulty mobilising, requiring the use of a frame. Facial diplegia | Demyelinating polyneuropathy | Covid-19 preceded GBS by 21 days | Nasogastric tube for feeding due to swallowing  difficulties, without respiratory dysfunction. Intravenous immunoglobulin (IVIG) 0.4  g/kg daily for 5 days. Neurorehabilitation | Cytoalbuminologic  dissociation (protein >1.25 g/L, white  cell count 1×106/L). His CSF was negative for  SARS-CoV-  2 | Absent sensory response of the  median nerve and severe slowing of  motor responses of the median and ulnar nerve  with reduced amplitude. Severe  neurogenic firing with normal motor units,  without spontaneous activity, on electromyography | Symptoms remarkably improved after neurorehabilitation |

| **Study** | **Sample size (number of male patients)** | **Age**  **(years)** | **GBS symptoms** | **GBS sub-type** | **Timing between Covid-19 symptoms onset and GBS onset (days)** | **Treatments received** | **CSF Examination** | **Electrophysiology** | **Outcome** |
| --- | --- | --- | --- | --- | --- | --- | --- | --- | --- |
| Farzi et al. (2020) | 1 (1) | 41 | Paraesthesia, absent muscle stretch reflexes in lower extremities and diminished reflexes  in upper extremities. Symmetric limb weakness and severe stocking-and-glove hypesthesia and  reduced vibration and position sense at all 4 limbs. Pain and an aching discomfort in the  muscles | Demyelination pattern polyneuropathy | Covid-19 preceded GBS by 10 days | IV immunoglobulins (0.4 g/kg per day for 5 consecutive  days) | Nil | Absence of right tibial and both  peroneal nerves compound muscle action potentials (CMAP). F waves  were unobtainable at lower limbs. Left tibial, Right and left median,  and ulnar nerves distal latencies were markedly prolonged and velocities  and amplitudes (with spatial and temporal dispersion) were decreased. | Patient improved remarkably and was referred for rehabilitation |

| **Study** | **Sample size (number of male patients)** | **Age**  **(years)** | **GBS symptoms** | **GBS sub-type** | **Timing between Covid-19 symptoms onset and GBS onset (days)** | **Treatments received** | **CSF Examination** | **Electrophysiology** | **Outcome** |
| --- | --- | --- | --- | --- | --- | --- | --- | --- | --- |
| Manganotti et al. (2020)^1^ | 1 (00) | 50 | Walking impairment due to ataxia,  ophthalmoplegia with diplopia in vertical and lateral gaze, left  upper arm cerebellar dysmetria, generalized areflexia, mild  lower facial defects, and mild hypoesthesia in the left mandibular  and maxillary branch of the face | Miller Fisher Syndrome | Covid-19 preceded GBS by 16days | Intravenous immunoglobulin (IVIG) therapy was initiated at  0.4 g/kg for 5 days | CSF protein concentration was  74.9 mg/dL, higher compared with normal values < 45 mg/  dL. | Nil | Neurological symptoms resolved and the patient was able to walk  without signs of ataxia. |

| **Study** | **Sample size (number of male patients)** | **Age**  **(years)** | **GBS symptoms** | **GBS sub-type** | **Timing between Covid-19 symptoms onset and GBS onset (days)** | **Treatments received** | **CSF Examination** | **Electrophysiology** | **Outcome** |
| --- | --- | --- | --- | --- | --- | --- | --- | --- | --- |
| Chan et al. (2020)^2^ | Case 1: 1 (1) | Case 1: 68 | Case 1: weakness in hip flexors and absent vibratory  and proprioceptive sense at the toes. Reduced reflexes, unsteady gait  was unsteady, bilateral facial weakness, dysphagia, dysarthria, neck flexion  weakness, and inability to ambulate. | Case 1: demyelination pattern polyneuropathy | Case 1: Covid-19 preceded GBS by 18 days | Case 1: five sessions of plasmapheresis | Case 1: WBC (Cells/mm3)=3, Protein (mg/dL)= 226, Glucose (mg/dL)= 56, Covid-19 PCR =negative | Case 1: Nil | Case 1: remarkable improved and was discharged to continue rehabilitation |

| **Study** | **Sample size (number of male patients)** | **Age**  **(years)** | **GBS symptoms** | **GBS sub-type** | **Timing between Covid-19 symptoms onset and GBS onset (days)** | **Treatments received** | **CSF Examination** | **Electrophysiology** | **Outcome** |
| --- | --- | --- | --- | --- | --- | --- | --- | --- | --- |
| Chan et al. (2020)^2^  (continued) | Case 2: 1 (1) | Case 2: 84 | Case 2: paresthesias of his hands and feet, progressive gait disturbance, diminished vibration and proprioception at the toes. Rredced reflexes and unable walk independently | Case 2: demyelination pattern polyneuropathy | Case 2: Covid-19 preceded GBS by 23 days | Case 2: plasmapheresis. mechanical ventilation.  IVig | Case 2: WBC (Cells/mm3)=1, Protein (mg/dL)=67, Glucose (mg/dL)= 58, Covid-19 PCR =negative | Case 2: Nil | Remained quadriparetic with  intermittent autonomic dysfunction, but is slowly being weaned from the ventilator |

| **Study** | **Sample size (number of male patients)** | **Age**  **(years)** | **GBS symptoms** | **GBS sub-type** | **Timing between Covid-19 symptoms onset and GBS onset (days)** | **Treatments received** | **CSF Examination** | **Electrophysiology** | **Outcome** |
| --- | --- | --- | --- | --- | --- | --- | --- | --- | --- |
| Naddaf et al. (2020) | 1 (0) | 58 | Bilateral paraparesis,  imbalance, and severe lower  thoracic pain without radiation, rapidly progressive  gait difficulty, areflexia | Acute sensorimotor demyelinating polyradiculoneuropathy | Covid-19 preceded GBS by 11 days | 5-day  course of hydroxychloroquine,  zinc, and methylprednisolone 40  mg twice daily for 5 days | A protein of 273 mg/dL  and 2 total nucleated cells  Negative for SARS COV-2 | Low  amplitude and prolonged duration of  the upper- and lower-limb compound  muscle-action potentials, with prolongation  of motor distal latencies, mild  slowing of motor conduction velocities,  and prolonged F-wave latencies. | Gait improved. Although she  remained slightly ataxic, she no longer  required a gait aid. |

| **Study** | **Sample size (number of male patients)** | **Age**  **(years)** | **GBS symptoms** | **GBS sub-type** | **Timing between Covid-19 symptoms onset and GBS onset (days)** | **Treatments received** | **CSF Examination** | **Electrophysiology** | **Outcome** |
| --- | --- | --- | --- | --- | --- | --- | --- | --- | --- |
| Mozhdehipanah et al. (2020) | Case 1: 1 (1) | 38 | Case 1: symmetric progressive ascending quardiparesthesia. bilateral facial droop, areflexia, decrease in all sensation  modalities in 4 limbs affecting the distal parts up to ankle and elbow  joints.  Romberg test was positive. | Case 1: acute demyelinating polyneuropathy | Case 1: Covid-19 preceded GBS by 21 days | Case 1: therapeutic plasma exchange  (TPE). labetalol by intravenous  bolus was administ | Case 1: Normal and negative for SARS COV-2 | Case 1: considerable reduction in the  compound motor action potentials amplitude with prolonged distal  latency and reduced conduction velocity . F and H waves were absent. | Case 1: Not reported  . |

| **Study** | **Sample size (number of male patients)** | **Age**  **(years)** | **GBS symptoms** | **GBS sub-type** | **Timing between Covid-19 symptoms onset and GBS onset (days)** | **Treatments received** | **CSF Examination** | **Electrophysiology** | **Outcome** |
| --- | --- | --- | --- | --- | --- | --- | --- | --- | --- |
| Mozhdehipanah et al. (2020)  (Continued) | Case 2: 1 (0) | Case 2: 14 | Case 2: progressive ascending quadripareshtesia, mild lower limb weakness, areflexia, decreased light touch, position, and vibration sensation in all distal  limbs up to ankle and elbow joints. Ataxic. | Case 2: acute demyelinating polyneuropathy | Case 2: Indeterminate | Case 2: intravenous immunoglobulin and ral hydroxychloroquine sulfate | Case 2: albuminocytologic dissociation | Case 2: Nil | Case 2: Patient eventually improved  . |

| **Study** | **Sample size (number of male patients)** | **Age**  **(years)** | **GBS symptoms** | **GBS sub-type** | **Timing between Covid-19 symptoms onset and GBS onset (days)** | **Treatments received** | **CSF Examination** | **Electrophysiology** | **Outcome** |
| --- | --- | --- | --- | --- | --- | --- | --- | --- | --- |
| Mozhdehipanah et al. (2020)  (Continued) | Case 3: 1 (0) | Case 3: 55 | Case 3: acute progressive lower limb weakness, Deep tendon reflexes were generally absent, and the sensory examination revealed decreased pinprick and vibration sensations in distal extremities. The patient was non-ambulatory | Case 3: acute demyelinating polyneuropathy | Case 3: Covid-19 preceded GBS by 26 days | Case 3: hydroxy chloroquine (HCQ; 200 mg twice a day for 10 days) and Kaletra (200/50 two tablets twice a day for 5 days). Intubation.  intravenous immunoglobulin (20 g IV daily for five days) | Case 3: average glucose, cell count, and protein (57 mg/dL protein) | Case 3: considerable reduction and absent CMAP amplitude with average distal latency and conduction velocity. The ulnar, F and H waves were absent. | Case 3: Patients eventually developed acute respiratory distress syndrome and died |

| **Study** | **Sample size (number of male patients)** | **Age**  **(years)** | **GBS symptoms** | **GBS sub-type** | **Timing between Covid-19 symptoms onset and GBS onset (days)** | **Treatments received** | **CSF Examination** | **Electrophysiology** | **Outcome** |
| --- | --- | --- | --- | --- | --- | --- | --- | --- | --- |
| Mozhdehipanah et al. (2020)  (Continued) | Case 4: 1 (0) | Case 4: 66 | Case 4: Progressive quadriparesis, Deep tendon reflexes were absent. Sensory examination revealed decreased light touch, position, and vibration sensation in all distal limbs up to ankle and elbow joints | Case 4: acute demyelinating polyneuropathy | Case 4: Covid-19 preceded GBS by 30 days | Case 4: Immunoglobulin (IVg) | Case 4: average glucose and cell count and 89 mg/dl protein | Case 4: a considerable reduction in the CMAP amplitude with prolonged distal latency and reduced conduction velocity. F and H waves were prolonged. | Case 4: She did not respond well to the treatment and eventually discharged to a rehabilitation facility for physical therapy  . |

| **Study** | **Sample size (number of male patients)** | **Age**  **(years)** | **GBS symptoms** | **GBS sub-type** | **Timing between Covid-19 symptoms onset and GBS onset (days)** | **Treatments received** | **CSF Examination** | **Electrophysiology** | **Outcome** |
| --- | --- | --- | --- | --- | --- | --- | --- | --- | --- |
| Embrahimzadeh et al. (2020) | Case1: 1 (1) | Case 1: 46 | Case 1: Mild peripheral facial nerve palsy on the right side. pain and numbness in distal lower and upper extremities, progressive ascending weakness in legs and areflexia. | Case 1: demyelinating polyneuropathy | Case 1: Covid-19 preceded GBS by 18 days | Case 1: Hydroxychloroquine for five days | Case 1: increased protein level (78 mg/dl, Normal:20-40 mg/dl) | Case 1: increased distal latencies in all  four extremities. There was also symmetric nerve conduction slowing | Case 1: After 16 days of close monitoring, his muscle forces  improved to near normal  . |

| **Study** | **Sample size (number of male patients)** | **Age**  **(years)** | **GBS symptoms** | **GBS sub-type** | **Timing between Covid-19 symptoms onset and GBS onset (days)** | **Treatments received** | **CSF Examination** | **Electrophysiology** | **Outcome** |
| --- | --- | --- | --- | --- | --- | --- | --- | --- | --- |
| Embrahimzadeh et al. (2020)  (continued) | Case 2: 1 (1) | Case 2: 65 | Case 2: a progressive ascending lower and upper extremities weakness and paresthesia. Areflexia. | Case 2: demyelinating polyneuropathy | Case 2: Covid-19 preceded GBS by 18 days | Case 2: IVG | Case 2: Nil | Case 2: generalized increased distal latencies and  absent F waves | Case 2: Muscle forces  improved and the patient was discharged  . |

| **Study** | **Sample size (number of male patients)** | **Age**  **(years)** | **GBS symptoms** | **GBS sub-type** | **Timing between Covid-19 symptoms onset and GBS onset (days)** | **Treatments received** | **CSF Examination** | **Electrophysiology** | **Outcome** |
| --- | --- | --- | --- | --- | --- | --- | --- | --- | --- |
| Pfefferkorn et al. (2020)^1^ | 1 (1) | 51 | Progressive upper  and lower limb weakness, acral paresthesias, generalized areflexia,  complete  peripheral locked-in syndrome with tetraplegia and sensory loss in all extremities, bilateral facial and hypoglossal  paresis and respiratory failure due to muscular  weakness | Acute demylinating polyneuropathy | Covid-19 preceded GBS by 12 days | Intravenous immunoglobulins  (IVIG, 30 g daily for 5 days, plasma  exchange therapy | Pleocytosis (9 cells/μl), normal protein content and negative  PCR testing for SARS-CoV-2. | Prolonged distal motor latencies (left median nerve  8.4 ms; left tibial nerve 11.6 ms) and loss of F waves | Patient was still on ventilator, but received specialized rehabilitation which resulted in improvement in symptoms |

| **Study** | **Sample size (number of male patients)** | **Age**  **(years)** | **GBS symptoms** | **GBS sub-type** | **Timing between Covid-19 symptoms onset and GBS onset (days)** | **Treatments received** | **CSF Examination** | **Electrophysiology** | **Outcome** |
| --- | --- | --- | --- | --- | --- | --- | --- | --- | --- |
| Gigli et al. (2020)^1^ | Case 1:1 (1)  Case 2:1 (1) | Case 1: 76  Case 2: 70 | Case 1:Tetraparesis  Dysarthria  Dysautonomia  Case 2:Paraparesis  Paraesthesia  Ataxia | Case 1:AMSAN  Case 2: AIDP | Case 1: Indeterminate  Case 2: Indeterminate | Case 1: Nil  Case 2: Nil | Case 1: CSF proteins =228 mg/L (*r*:150–450 mg/L),  CSF leucocytes=0.6/μL  (*r*: 0–3/μL  Case 2: CSF proteins = 216 mg/L (*r*:150–450 mg/L), CSF leucocytes= 0.6/μL(*r*: 0–3/μL) | Case 1: signs of AMSAN  Case 2: signs of AIDP | Case 1: Nil  Case 2: Nil |

| **Study** | **Sample size (number of male patients)** | **Age**  **(years)** | **GBS symptoms** | **GBS sub-type** | **Timing between Covid-19 symptoms onset and GBS onset (days)** | **Treatments received** | **CSF Examination** | **Electrophysiology** | **Outcome** |
| --- | --- | --- | --- | --- | --- | --- | --- | --- | --- |
| Gigli et al. (2020)  (continued) | Case 3:1 (1)  Case 4:1 (1) | Case 3: 80  Case 4: 59 | Case 3: Arthromyalgia  Low back pain  Paraesthesia  Paraparesis  Case 4: Emifacial paresthesia, Facial weakness (CN VII)  Dysarthria (CN XII) | Case 3: AIDP  Case 4: Altered blink reflex, demyelinating  damage (MFS) | Case 3: Indeterminate  Case 4: Indeterminate | Case 3: Nil  Case 4: Nil | Case 3: CSF proteins = 933 mg/L (*r*:150–450 mg/L), CSF leucocytes= 0/μL (*r*: 0–3/μL)  Case 4: CSF proteins = 701 mg/L (*r*:150–450 mg/L), CSF leucocytes= 2.8/μL (*r*: 0–3/μL) | Case 1: signs of AIDP  Case 4: signs of MFS | Case 1: Nil  Case 4: Nil |

| **Study** | **Sample size (number of male patients)** | **Age**  **(years)** | **GBS symptoms** | **GBS sub-type** | **Timing between Covid-19 symptoms onset and GBS onset (days)** | **Treatments received** | **CSF Examination** | **Electrophysiology** | **Outcome** |
| --- | --- | --- | --- | --- | --- | --- | --- | --- | --- |
| Gigli et al. (2020)  (continued) | Case 5:1 (0)  Case 6: 1(1) | Case 5: 59  Case 6: 82 | Case 5: Low back pain  Paraesthesia  Tetraparesis  Case 6: Asymmetric paraparesis | Case 5: AIDP  Case 6: AIDP | Case 5: Indeterminate  Case 6: Indeterminate | Case 5: Nil  Case 6: Nil | Case 5: CSF proteins = 1124 mg/L (*r*:150–450 mg/L), CSF leucocytes= 0.4/μL (*r*: 0–3/Μl  Case 6: CSF proteins = 827 mg/L (*r*:150–450 mg/L), CSF leucocytes= 0.8/μL (*r*: 0–3/μL | Case 5: signs of AIDP  Case 6: signs of AIDP | Case 5: Nil  Case 6: Nil |

| **Study** | **Sample size (number of male patients)** | **Age**  **(years)** | **GBS symptoms** | **GBS sub-type** | **Timing between Covid-19 symptoms onset and GBS onset (days)** | **Treatments received** | **CSF Examination** | **Electrophysiology** | **Outcome** |
| --- | --- | --- | --- | --- | --- | --- | --- | --- | --- |
| Gigli et al. (2020)  (continued) | Case 7: 1(1)  Case 8: 1(0) | Case 7: 53  Case 8: 59 | Case 7: Paraesthesia  Ataxia  Case 8: Tetraparesis  Paraesthesia | Case 7: AIDP  Case 8: AIDP | Case 7: Indeterminate  Case 8: Indeterminate | Case 7: Nil  Case 8: Nil | Case 7: CSF proteins = 1928 mg/L (*r*:150–450 mg/L), CSF leucocytes= 2.6/μL (*r*: 0–3/μL  Case 8: Relapse (not applicable) | Case 7: signs of AIDP  Case 8: signs of AIDP | Case 7: Nil  Case 8: Nil |

| **Study** | **Sample size (number of male patients)** | **Age**  **(years)** | **GBS symptoms** | **GBS sub-type** | **Timing between Covid-19 symptoms onset and GBS onset (days)** | **Treatments received** | **CSF Examination** | **Electrophysiology** | **Outcome** |
| --- | --- | --- | --- | --- | --- | --- | --- | --- | --- |
| Sidig et al. (2020) | 1 (1) | 65 | Quadriplegia and numbness, facial  paraesthesia with the inability to close his mouth, and his both eyes, clumsiness of both upper limbs, urinary incontinence, progressive paralysis, facial paralysis, truncal weakness, generalized hypotonia and areflexia | Polyradiculopathy | Covid-19 preceded GBS by 5 days | IV immunoglobulin and mechanical ventilator | Nil | Evidence of  polyradiculopathies | Not reported |

| **Study** | **Sample size (number of male patients)** | **Age**  **(years)** | **GBS symptoms** | **GBS sub-type** | **Timing between Covid-19 symptoms onset and GBS onset (days)** | **Treatments received** | **CSF Examination** | **Electrophysiology** | **Outcome** |
| --- | --- | --- | --- | --- | --- | --- | --- | --- | --- |
| Fernandes-Dominguez et al. (2020) | 1 (0) | 74 | Progressive  gait impairment.  Lower limbs areflexia  with patent gait ataxia. | Miller Fisher Like syndrome | Covid-19 preceded GBS by 12-15 days | Hydroxychloroquine  and lopinavir/ritonavir, | Intravenous immunoglobulins. CSF SARS-CoV-2 PCR was also negative | Slight F-wave delay in  upper limbs | An improvement  in gait was observed, and also, lower limb reflexes  were slightly present. |

| **Study** | **Sample size (number of male patients)** | **Age**  **(years)** | **GBS symptoms** | **GBS sub-type** | **Timing between Covid-19 symptoms onset and GBS onset (days)** | **Treatments received** | **CSF Examination** | **Electrophysiology** | **Outcome** |
| --- | --- | --- | --- | --- | --- | --- | --- | --- | --- |
| Paterson et al. (2020) | 7 (7) | 20-63 | One patient developed brachial plexopathy | Not reported | Covid-19 preceded GBS by 1 to 21 days | All received IVIG.  The patient with brachial plexopathy  received corticosteroids | Not reported | .Not reported | All but two of this group have started to make partial recovery |

| **Study** | **Sample size (number of male patients)** | **Age**  **(years)** | **GBS symptoms** | **GBS sub-type** | **Timing between Covid-19 symptoms onset and GBS onset (days)** | **Treatments received** | **CSF Examination** | **Electrophysiology** | **Outcome** |
| --- | --- | --- | --- | --- | --- | --- | --- | --- | --- |
| Manganotti^2^ al. (2020) | Case 1: 1(1)  Case 2: 1(1) | Case 1: 72  Case 2: 72 | Case 1: Flaccid tetraparesis with proximal upper limb predominance, diffused arefexia, tingling sensation in the distal lower limb, lower right sided facial weakness  Case 2: Flaccid tetraparesis with proximal upper limb predominance, diffused arefexia and sense of having a tight bandage on legs and feet | Case 1: Acute demylinating polyneuropathy  Case 2: Acute demylinating polyneuropathy | Case 1: Covid-19 preceded GBS by 18 days  Case 2: Covid-19 preceded GBS by 30 days | Case 1: IVIG cycle (0.4g/kg for 5 days).  Hydroxychloroquine, osetamivir, darunavir, methylpredinisolone and tocilizumab  Case 2: IVIG cycle (0.4g/kg for 5 days).  Hydroxychloroquine, Lopinavir-ritonavir, methylpredinisolone | Case 1: CSF proteins = 52 mg/dL; 1cell/mm^3^. Negative for Covid-19  Case 2: CSF proteins = 40 mg/dL; 1 cell/mm^3^. Negative for Covid-19 | All cases: Either an increase in latency of F wave  or the dispersion and the decrease in amplitude of F wave | Case 1: Improvement in tetraparesis  Case 2: Improvement in muscle weakness |

| **Study** | **Sample size (number of male patients)** | **Age**  **(years)** | **GBS symptoms** | **GBS sub-type** | **Timing between Covid-19 symptoms onset and GBS onset (days)** | **Treatments received** | **CSF Examination** | **Electrophysiology** | **Outcome** |
| --- | --- | --- | --- | --- | --- | --- | --- | --- | --- |
| Manganotti^2^ al. (2020)  (continued) | Case 3: 1(0)  Case 4: 1(1) | Case 3: 49  Case 4: 94 | Case 3: opthalmoplegia with diplopia in the vertical and lateral gaze, limb ataxia, diffused areflexia, right side hypoesthesia of the face.  Case 4: Lower limb weakness, diffused weak reflex on legs and feet | Case 3: Acute demylinating polyneuropathy  Case 4: Acute demylinating polyneuropathy | Case 3: Covid-19 preceded GBS by 14 days  18 days  Case 4: Covid-19 preceded GBS by 33 days | Case 3: IVIG cycle (0.4g/kg for 5 days).  Hydroxychloroquine, Lopinavir-ritonavir, methylpredinisolone  Case 4: methylpredinisolone 60mg for 5 days | Case 3: CSF proteins = 72 mg/dL; 5 cell/mm^3^. Negative for Covid-19  Case 4: Not performed | All cases: Either an increase in latency of F wave  or the dispersion and the decrease in amplitude of F wave | Case 3: Progressive improvement  Case 4: Stationary |

| **Study** | **Sample size (number of male patients)** | **Age**  **(years)** | **GBS symptoms** | **GBS sub-type** | **Timing between Covid-19 symptoms onset and GBS onset (days)** | **Treatments received** | **CSF Examination** | **Electrophysiology** | **Outcome** |
| --- | --- | --- | --- | --- | --- | --- | --- | --- | --- |
| Manganotti^2^ al. (2020)  (continued) | Case 5: 1(1) | Case 5: 76 | Case 5: proximal weakness of lower and upper limb, with upper limb predominance, diffused areflexia, left sided lower facial deficit, mild transient diplopia | Case 5: Acute demylinating polyneuropathy | Case 5: Covid-19 preceded GBS by 22 days | Case 5: IVIG cycle (0.4g/kg for 5 days).  Hydroxychloroquine, osetamivir, darunavir, methylpredinisolone and tocilizumab  Methylpredinisolone, meropenem, linezolid, clarithromycin, doxycycline and fluconazole | Case 5: CSF proteins = 53 mg/dL; 2 cell/mm^3^. Negative for Covid-19 | All cases: Either an increase in latency of F wave  or the dispersion and the decrease in amplitude of F wave | Case 5: Progressive improvement |
